# ATAD2 is an epigenetic reader of newly synthesized histone marks during DNA replication

**DOI:** 10.18632/oncotarget.11855

**Published:** 2016-09-06

**Authors:** Seong Joo Koo, Amaury E. Fernández-Montalván, Volker Badock, Christopher J. Ott, Simon J. Holton, Oliver von Ahsen, Joern Toedling, Sarah Vittori, James E. Bradner, Mátyás Gorjánácz

**Affiliations:** ^1^ Drug Discovery, Bayer Pharma AG, Berlin, Germany; ^2^ Center for the Science of Therapeutics, Broad Institute, Cambridge, MA, USA; ^3^ Department of Medical Oncology, Dana-Farber Cancer Institute, Boston, MA, USA; ^4^ Department of Medicine, Harvard Medical School, Boston, MA, USA; ^5^ Present address: Novartis Institute for BioMedical Research, Cambridge, MA, USA

**Keywords:** cancer, ATAD2, DNA replication, bromodomain, histone acetylation

## Abstract

ATAD2 (ATPase family AAA domain-containing protein 2) is a chromatin regulator harboring an AAA+ ATPase domain and a bromodomain, previously proposed to function as an oncogenic transcription co-factor. Here we suggest that ATAD2 is also required for DNA replication. ATAD2 is co-expressed with genes involved in DNA replication in various cancer types and predominantly expressed in S phase cells where it localized on nascent chromatin (replication sites). Our extensive biochemical and cellular analyses revealed that ATAD2 is recruited to replication sites through a direct interaction with di-acetylated histone H4 at K5 and K12, indicative of newly synthesized histones during replication-coupled chromatin reassembly. Similar to ATAD2-depletion, ectopic expression of ATAD2 mutants that are deficient in binding to these di-acetylation marks resulted in reduced DNA replication and impaired loading of PCNA onto chromatin, suggesting relevance of ATAD2 in DNA replication. Taken together, our data show a novel function of ATAD2 in cancer and for the first time identify a reader of newly synthesized histone di-acetylation-marks during replication.

## INTRODUCTION

Epigenetic mechanisms play essential roles during the cell cycle and following cell division to maintain gene expression control and cell state. Epigenetic modifications include methylation of DNA and post-translational modifications of proteins that influence gene expression by serving as a signaling platform for chromatin modifying enzymes and associated effector proteins [1].

An important post-translational modification of histone proteins is the acetylation of lysines. Generally, these acetylation marks are associated with euchromatin architecture and active transcription [1]. However, recent advances have revealed that acetylation marks on histones are also involved in chromatin compaction [2], DNA repair [3] and DNA replication [4]. For example, during replication-coupled nucleosome assembly, newly synthesized histone H4 proteins are transiently acetylated at lysine 5 and 12, a phenomenon conserved through evolution from yeast to mammals [5, 6]. Supporting the importance of histone acetylation during DNA replication, a recent study showed that a small molecule inhibitor of histone deacetylases (HDACs) led to perturbed DNA replication [7]. Yet, the ‘readers’ of these acetylation marks and their molecular functions during DNA replication and chromatin reassembly remain unknown.

One of the best characterized ‘readers’ of acetylation marks are bromodomain (BD)-containing proteins. BDs directly interact with acetylated lysines through a conserved asparagine in a hydrophobic pocket [8, 9]. ATAD2 (ATPase family AAA domain-containing protein 2) is one such BD-containing protein. Through a conserved binding mode the BD of ATAD2 recognizes histone H3K9,14ac [10] or histone H4K5ac [11]. Unlike other proteins of the family, ATAD2 requires multimerization via its ATPase domain for the BD function [11]. In cancer cells ATAD2 has been proposed to function as a transcriptional co-regulator of several oncogenic transcriptional factors including estrogen receptor (ER) [12], androgen receptor (AR) [13], E2F transcriptional factor [10] and Myc [14]. Recently, a number of reports have demonstrated that expression of ATAD2 strongly correlates with poor prognosis in different unrelated tumors including gastric cancer [15], endometrial carcinoma [16], hepatocellular carcinoma [17], ovarian carcinoma [18], breast cancer [11, 19] and lung cancer [11] and hence proposed ATAD2 as a poor prognostic marker. Yet it is unclear whether the transcriptional co-regulator function of ATAD2 is the predominant mechanism of malignancy in tumors of diverse origins.

In this study we describe novel molecular mechanisms that define a new role for ATAD2 in cancer cell proliferation. We found that during DNA replication ATAD2 is recruited to replication sites through its direct interaction with newly synthesized histones. Our study identified the first “reader” protein of these histone marks and provides evidence for a direct role of a bromodomain-containing protein in DNA replication. This mechanism may explain the reported strong association of ATAD2 expression levels with poor prognosis in various cancer types.

## RESULTS

### ATAD2 is expressed in S phase where it localizes to replication sites

**Figure 1:**
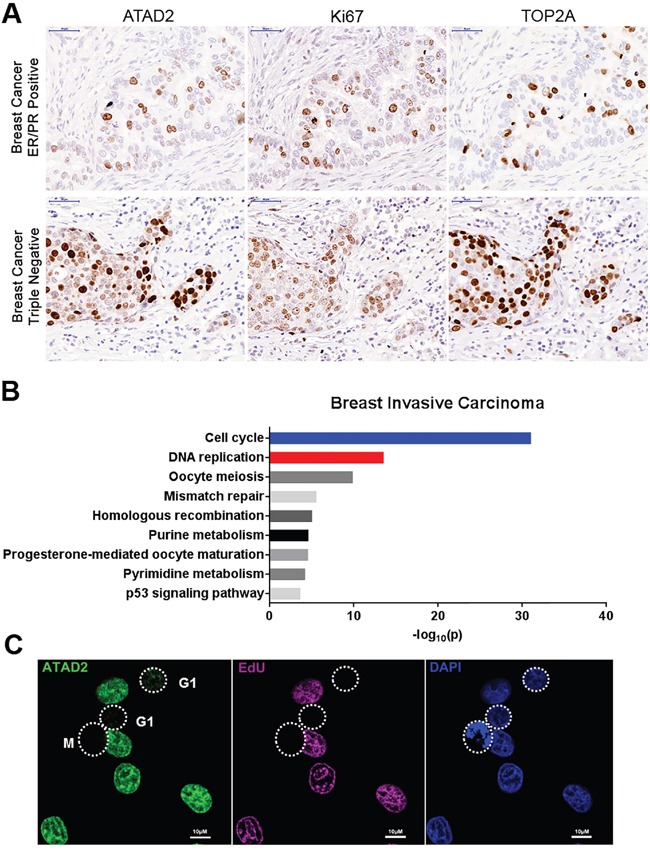
Expression of ATAD2 in primary tumors and MCF7 breast cancer cells **A.** ATAD2 expression is restricted to areas positive for Ki67 and TOP2A in ER/PR positive and triple negative breast cancer. Consecutive tissue microarray (TMA) sections from breast cancer were stained with ATAD2, Ki67 and TOP2A by immunohistochemistry. 40 TMA cores of independent tumor samples were analyzed. Representative images are shown here. (scale bar: 50 μm) **B.** Cell cycle and DNA replication related genes are positively correlated with ATAD2 gene expression. Genes that are co-expressed with ATAD2 in breast invasive carcinoma were identified from 960 samples and pathways in which these genes function were examined. −log_10_(p) are plotted here. **C.** ATAD2 is expressed in S phase cells. MCF7 cells were fixed in 4 % formaldehyde and stained with antibody against ATAD2 (green). Cells in S phase were visualized by incorporation of EdU into newly synthesized DNA (purple). Cells in M phase were identified by condensed DNA. DNA was stained with DAPI (blue). (scale bar: 10 μm; M: Mitosis) See also Figure S1.

In order to confirm the expression of ATAD2 in various tumors [11, 16–19], we performed immunohistochemistry analysis on tissue microarrays (TMA) from multiple cancer types including breast, prostate, gastric, colorectal and lung cancers (Figures 1A and Supplementary Figure S1A). ATAD2 was expressed in all tested tumors independent of their tissue of origin. Interestingly, ATAD2 expression was strongly restricted to the proliferating area of each tumor, as marked by Ki67 and Topoisomerase 2A (TOP2A) staining, suggesting that ATAD2 might be implicated in cell proliferation and cell cycle progression (Figures 1A and Supplementary Figure S1A). To test this possibility we interrogated gene expression datasets from The Cancer Genome Atlas (TCGA; Figures 1B and Supplementary Figure S1B). First we identified the genes that positively correlate with ATAD2 expression (Spearman score higher than 0.5) and determined the cellular pathways in which these genes function. ATAD2 expression positively correlated with genes involved in cell cycle and DNA replication. We note that this correlation is absent in prostate adenocarcinoma (Supplementary Figure S1B), likely due to the slow growth of this tumor type as evident by rare Ki67 positive cells (Supplementary Figure S1A). Taken together, these unbiased and systematic analyses in many unrelated tumors suggest that ATAD2 might function during DNA replication in addition to its previously reported role as a transcriptional cofactor of oncogenes.

**Figure 2:**
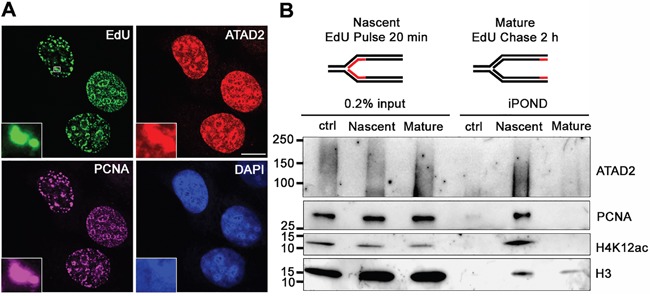
ATAD2 is recruited to DNA replication sites **A.** Co-localization of ATAD2 (red) with newly synthesized DNA (EdU, green) and PCNA (purple) in replication foci of MCF7 cells. Insets show an enlargement of the boxed area by 6 times. (scale bar: 10 μm) **B.** ATAD2 is recruited to nascent chromatin but not to mature chromatin. Proteins bound on nascent and mature chromatin were isolated by iPOND technology and analyzed by Western blotting. Ctrl: no addition of biotin azide. Smear band of ATAD2 is due to crosslinking of ATAD2 to chromatin and other proteins. See also Figure S2.

To test this hypothesis we first asked whether in cancer cell lines ATAD2 expression was also cell cycle-dependent as seen in clinical tumor samples (Figures 1A and 1B, Supplementary Figure S1A and B). In MCF7 cells ATAD2 was only detectable at S phase, as indicated by incorporation of the nucleotide analogue EdU (5-ethynyl-2'-deoxyuridine) during DNA replication in those cells. ATAD2 expression was significantly lower or absent at G1 and M phases (Figure 1C). We also confirmed the S phase-specific expression of ATAD2 by flow cytometry using three independent antibodies (Supplementary Figure S2B). We next asked whether ATAD2 is associated with DNA replication sites. ATAD2 colocalized with newly incorporated DNA (EdU) and the essential DNA replication protein PCNA (Figure 2A) at replication foci in MCF7 cells and in several other breast cancer cell lines (T47D, BT-549 and MDA-MB-231; Supplementary Figure S2A) independent of their hormone dependency. This observation was further confirmed biochemically using the iPOND technology (Isolation of Proteins On Nascent DNA [20]) (Figure 2B). Proteins bound on nascent chromatin were isolated by capturing newly synthesized DNA (incorporated EdU for 20 min at 37 °C) and analyzed by Western blot. To compare the proteins on nascent and mature chromatin, samples of mature chromatin were collected from cells chased for 2 hours after a 20 min pulse to allow for the post-replicative maturation of chromatin. ATAD2 was clearly detected on nascent chromatin together with PCNA and newly deposited histone H4 proteins reported to be acetylated at K12. However, ATAD2 was no longer detected on post-replicative chromatin, suggesting a function of ATAD2 at DNA replication sites.

### Depletion of ATAD2 or overexpression of dominant negative ATAD2 mutants abrogates DNA replication

**Figure 3:**
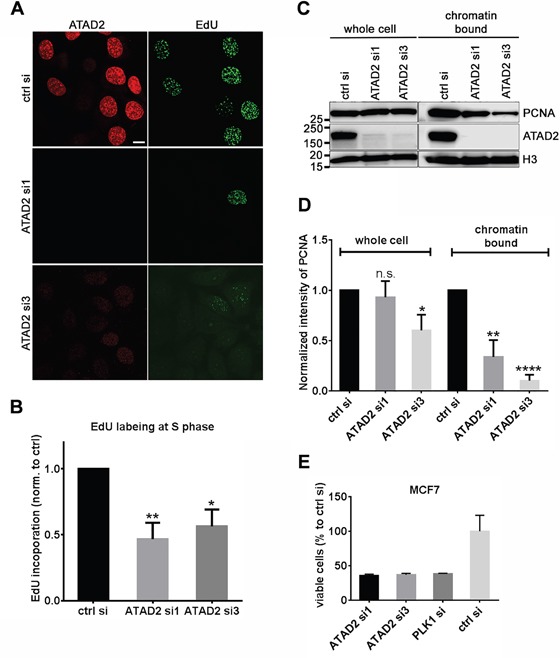
Depletion of ATAD2 in MCF7 cells impairs DNA replication **A.** and **B.** Reduced EdU incorporation in MCF7 cells depleted of ATAD2. 3 days after transfection with siRNAs targeting ATAD2 (ATAD2 si1 and ATAD2 si3) and control siRNA (ctrl si), MCF7 cells were incubated with 10 μM EdU for 2 h at 37 °C and directly fixed and processed for immunofluorescence (A) or flow cytometry (B). (B) Incorporated EdU during S phase, defined by DNA content, was quantified by flow cytometry. Data are represented as mean ± SEM. ATAD2 si1 = 0.47 ± 0.12 and ATAD2 si3 = 0.57 ± 0.13 (normalized to control; n = 4 independent experiments) **C.** and **D.** Reduced chromatin bound PCNA proteins in ATAD2-depleted MCF7 cells in comparison to control cells. Chromatin bound proteins were solubilized by incubation with MNase for 5 min at 37 °C and analyzed in Western blotting (C) and represented by a bar graph (D) by mean ± SEM. In “whole cell” ATAD2 si1 = 0.83 ± 0.16 and ATAD2 si3 = 0.60 ± 0.16 and in “chromatin bound” ATAD2 si1 = 0.34 ± 0.17 and ATAD2 si3 = 0.10 ± 0.06 (normalized to control; n = 4 independent experiments; student *t*-test *P < 0.05; **P < 0.01; ***P < 0.001; ****P < 0.0001) **E.** Slower cell growth upon depletion of ATAD2 in MCF7 cells. 4 days after transfection with siRNAs viable cells were measured by AlamarBlue. Fluorescence intensity was normalized to control siRNA treated cells. siRNA1 = 35.9 % ± 2.0, siRNA3 = 37.0 % ± 2.0 and PLK1 = 38.5 % ± 0.6 (mean ± SD, n = 3) See also Figure S3.

We next addressed whether DNA replication would be impaired by depletion of endogenous ATAD2 or ectopic expression of mutant ATAD2. Following depletion of ATAD2 in MCF7 cells by two siRNAs targeting ATAD2, we observed a reduction of EdU incorporation when compared to control siRNA treated cells (Figure 3A). We also quantitatively analyzed DNA replication by flow cytometry and observed the significant reduction of DNA replication in cells depleted of ATAD2 (Figure 3B). Only cells in S phase were considered for analysis to avoid possible indirect consequences due to cell cycle arrest. Interestingly, in these cells we found that the chromatin bound fraction of PCNA was drastically reduced, to 34 % for siRNA1 and 10 % for siRNA3 compared to control siRNA-treated cells, while the total cellular content of PCNA was not significantly altered (Figure 3C and 3D). Consequently, proliferation of ATAD2-depleted MCF7 cells was strongly decreased to 37 % of controls, comparable to polo-like kinase 1 (PLK1) knockdown (Figure 3E). We confirmed the proliferation inhibition upon ATAD2 depletion in other breast cancer cell lines including T47D, BT-549 and MDA-MB-231 (Supplementary Figure S3).

**Figure 4:**
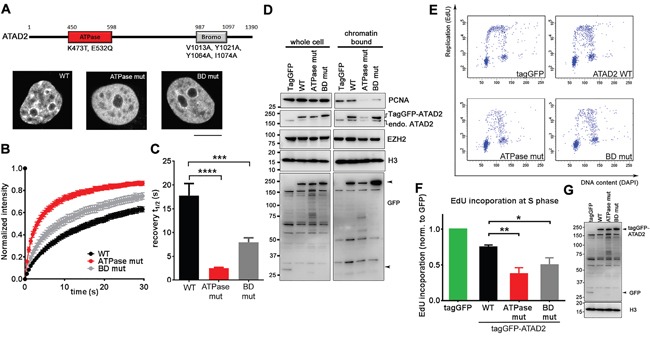
Overexpression of ATAD2 mutants incapable of chromatin binding leads to perturbed DNA replication **A.** TagGFP-tagged WT (WT) ATAD2, ATPase mutant (ATPase mut) and BD mutant (BD mut) ATAD2 both defective of acetylated histone binding were expressed in MCF7 cells and images were taken from live cells by a confocal microscope. **B.** A circular area with a diameter of 2.8 μm in nucleus were bleached by a laser at 100 % power and recovery of fluorescence was monitored every 486 msec for 70 images. **C.** Half recovery time (recovery t_1/2_) calculated from 20 images is plotted in a bar graph; WT = 17.740 sec ± 2.567, ATPase mut = 2.514 sec ± 0.156 and BD mut = 7.951 sec ± 0.950 (mean ± SEM). **D.** Reduced recruitment of PCNA onto chromatin upon mutant ATAD2 expression. 16 h after transfection with tagGFP-tagged WT and mutant ATAD2 in MCF7 cells were subjected for subcellular fractionation and Western blotting. Arrowheads indicate tagGFP and tagGFP-tagged ATAD2. **E. F.** and **G.** Overexpression of mutant ATAD2 abrogates DNA replication. **E.** WT or mutant ATAD2 expressing cells that were gated by their tagGFP expression in flow cytometry are plotted based on EdU and DAPI. **F.** Quantification of EdU incorporation; WT = 0.75 ± 0.03, ATPase mut = 0.38 ± 0.09 and BD mut = 0.5 ± 0.10. (normalized to control; mean ± SEM, n = 3 independent experiments) **G.** Cell lysates from tagGFP, tagGFP-tagged WT and mutant ATAD2 were resolved on SDS-PAGE followed by Western blotting using antibodies against GFP. The result confirms the overexpression of each constructs. Student *t*-test; *P < 0.05; **P < 0.01; ***P < 0.001.

In order to strengthen our observations, and to gain insights into molecular mechanism we examined mutagenized ATAD2 in DNA replication. ATAD2 is comprised of an AAA+ ATPase domain and a Bromodomain (BD) both of which have been shown to be essential for acetylated histone interaction (Figure 4A; [10, 14, 21]). We generated an ATPase inactive mutant ATAD2 (ATPase mut; K473T and E532Q) and BD mutant ATAD2 (V1013A, Y1021A, Y1064A and I1074A) that lacks the ability to bind to acetylated histones [10, 14] and tested their recruitment to chromatin by FRAP (fluorescence recovery after photobleaching; Figures 4A – 4C). Sub-cellular localization of ectopically expressed tagGFP-tagged WT ATAD2 was similar to endogenous ATAD2 (Figure 2A) while both ATPase and BD mutant ATAD2 proteins were more diffuse in the nucleus and were no longer localized to nuclear speckles (Figure 4A), implying compromised recruitment to chromatin. In line with this observation half fluorescence recovery time after photobleaching (recovery t_1/2_) of these mutant ATAD2 proteins was significantly faster than of the WT (Figure 4B and 4C), confirming that these mutants are not tightly bound to chromatin compared to the WT. Expressing these mutant ATAD2 proteins in MCF7 cells resulted in less PCNA association with chromatin (Figure 4D) and significant reduction in DNA replication (Figures 4E, 4F and 4G), recapitulating the phenotypes that we observed in ATAD2-depleted cells. Taken together, these results suggest that the recruitment of ATAD2 to chromatin mediated by the ATPase- and bromodomain is important for DNA replication.

### Identification of ATAD2 interacting proteins through acetylation

**Figure 5:**
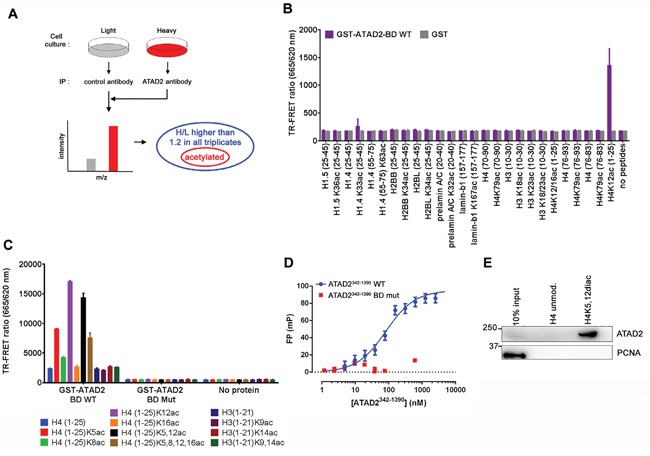
ATAD2 interacts with Histone 4 acetylated at K5 and K12 **A.** Outline of SILAC experiment to identify ATAD2 interacting proteins through acetylation modification. Immunoprecipitation was carried out using control antibody and antibody against ATAD2 on MCF7 cells grown in light (L) and heavy (H; K8, R10) medium, respectively and bound proteins were analyzed by mass spectrometry. Unmodified as well as acetylated peptides were identified. Only the ones with log_10_ of the SILAC ratio H/L higher than 1.2 in all triplicates were considered as hits (Figure S4). **B.** Among the hits H4 (1-25) peptides with acetylation at K12 showed a strong interaction with BD of ATAD2. 1 μM of Biotin-labelled peptides with acetylation identified in the SILAC experiment (A and S4) were incubated with 10 nM of GST-ATAD2 BD (GST-ATAD2-BD WT) or GST as a control and the interaction was monitored by TR-FRET technology. **C.** H4 (1-25) peptides acetylated at K5, K12, K5,12 or K5,8,12,16 interact with WT BD (GST-ATAD2-BD WT) but not with mutant BD (GST-ATAD2-BD Mut) in the same TR-FRET assay. **D.** An FP titration of ATAD2^342-1390^ WT (blue) or BD mutant protein (red) at the concentrations indicated on 8 nM H4K12ac-TAMRA peptide yielded binding saturation curves which in the case of the WT protein could be fitted (solid line) to a one site specific binding model resulting in a K_D_ value of 85 ± 15 nM. **E.** Endogenous ATAD2 interacts with H4K5,12diac (1-25) but not with unmodified H4 (1-25). Immobilized histone peptides were incubated with nuclear extract from MCF7 cells and bound proteins were analyzed by Western blotting. See also Figures S5 and S6 and SI S4.

Given that mutations within the bromodomain binding interface with acetylated proteins cause replication defects, we hypothesized that ATAD2 functions at replication sites through its interaction with acetylated proteins. Therefore we searched for proteins that interact with ATAD2 via acetylation by performing stable isotope labelling of amino acids in cell culture (SILAC)-based proteomic analysis of ATAD2 associated proteins (Figure 5A). First we identified proteins that were enriched in ATAD2-specific immunoprecipitation samples with log_10_ H/L ratio higher than 1.2 across three independent replicates. Most peptides corresponded to histone H3 (HIST1H3A;HIST3H3;H3F3C; log_10_ H/L ratio = 2.1, average) and H4 (HIST1H4A; log_10_ H/L ratio = 2.1, average; Supplementary Figure S4), providing additional evidence that these two proteins are the main interacting partners of ATAD2. Next, we examined the acetylation status of peptides corresponding to ATAD2 interacting proteins and found that the peptides from histones including H1.4, H1.5, H2, H3 and H4 as well as non-histone proteins such as prelamin A/C and lamin B1 were acetylated. To confirm these interactions we obtained 20-25 mer peptides with the acetylation modification in the middle of the peptides (Supplemental Experimental Procedures) and tested their interaction with the BD of ATAD2 by TR-FRET (Time-Resolved Fluorescence Energy Transfer) using GST-tagged WT ATAD2-BD (amino acid 981 – 1108) and GST as a control (Figure 5B). While H4K12ac peptides showed strong interaction with ATAD2-BD other peptides exhibited no binding. Next, relative binding affinity of the H4K12ac peptide was compared to other acetylated histone peptides that were previously identified to interact with ATAD2 [10, 11] (Figure 5C). We observed strong binding of ATAD2-BD to histone H4 peptides acetylated at K12, as well as at K5, whereas histone H3 peptides displayed no interaction at the concentrations tested. Importantly, these interactions were specific and likely involve the conserved BD-acetylated histone binding mode, because the binding was completely abolished by mutation of key acetyl-lysine binding residues within the ATAD2-BD (BD mut; Figure 5C and 5D; [10, 11]), the same mutations that we used in the FRAP and replication experiments (Figure 4). Further insight into recognition of the histone mark by ATAD2 was delivered by comprehensive biochemical analyses (MicroScale Thermophresis, Supplementary Figure S5A; Isothermal Calorimetry, Supplementary Figure S5B; TR-FRET competition assay, Supplementary Figure S5C; Surface Plasmon Resonance, Supplementary Figure S5D and HSQC binding site mapping Supplementary Figure S5E). While these studies showed micromolar affinity of the interaction of ATAD2 BRD the H4K12ac peptide, in a fluorescence polarization (FP) assay using an ATAD2 construct in which the ATPase domain the Kd for the binding was in the nanomolar range (Figure 5D).

### ATAD2 is recruited to replication sites with newly synthesized histones

Previously it has been reported that di-acetylation modification at K5 and K12 on histone H4 that we identified to interact with the recombinant bromodomain of ATAD2 (Figure 5C) occurs on newly synthesized histones during DNA replication. Intrigued by the idea that ATAD2 could be recruited to the replication sites via interaction with the di-acetylation mark we tested whether this modification on histone H4 is required for binding to endogenous ATAD2. In line with the TR-FRET experiment histone peptides (1-25) di-acetylated at K5 and K12 efficiently pulled down endogenous ATAD2 from nuclear extract prepared from MCF7 whereas the same peptides without the modification showed no interaction (Figure 5E).

**Figure 6:**
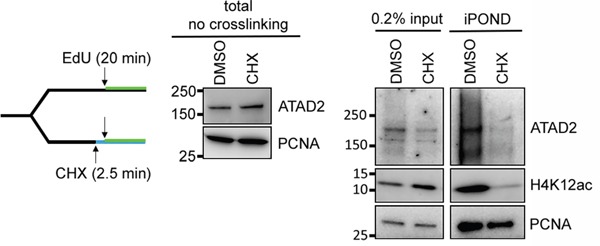
ATAD2 is recruited to replication sites by binding to newly synthesized histones Prevention of new histone deposition by cycloheximide (CHX) abrogated recruitment of ATAD2 to nascent chromatin. HEK293 cells were treated with 250 μg/ml of CHX for 2.5 min before the addition of EdU at 10 μM for 20 min at 37 °C (left). Cells were immediately lysed and total level of ATAD2 and PCNA before crosslinking was examined by Western blotting (middle) and nascent chromatin was isolated by iPOND and analyzed (right).

Next we asked whether the interaction of ATAD2 and the new histone marks is indeed required for the recruitment of ATAD2 to replication sites. To this aim we prevented the deposition of new histones on nascent chromatin by blocking histone synthesis with applying cycloheximide (CHX) for 2.5 min after which we analyzed the proteins on nascent chromatin by iPOND (Figure 6 left). In CHX-treated samples the newly synthesized histone H4 acetylated at K12 was lost from nascent chromatin, as previously shown [22]. Similarly, ATAD2 was also lost from the nascent chromatin (Figure 6 right), although the total level of ATAD2 was unaffected (Figure 6 middle). This result strongly supports that ATAD2 is recruited to the replication sites through its interaction with newly synthesized histone that involves the bromodomain of ATAD2 and di-acetylation of histone H4.

### ATAD2 inhibits new histones from interacting with HDAC1

**Figure 7:**
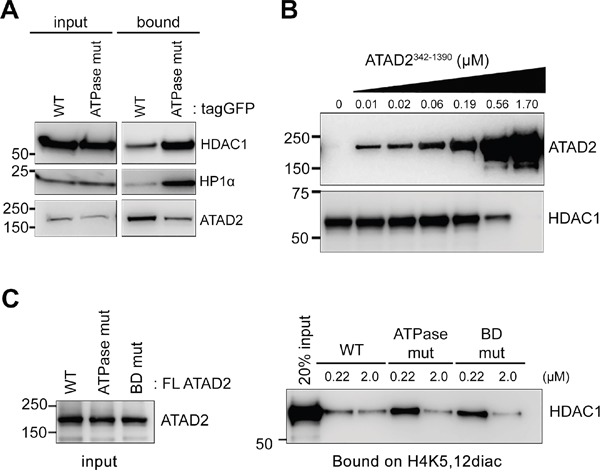
ATAD2 competes with HDAC1 for binding to H4K5,12diac **A.** Less histone deacetylase (HDAC) 1 was bound to H4K5,12diac (1-25) in presence of tagGFP-tagged WT ATAD2 in comparison to ATPase mutant ATAD2 (ATPase mut). Immobilized H4K5,12diac (1-25) was incubated with nuclear extract of MCF7 cells expressing either WT ATAD2 or ATPase mutant ATAD2 overnight and bound proteins were analyzed in Western blotting. **B.** Increasing amount of ATAD2 outcompetes HDAC1 for binding to the H4K5,12diac. 1 μM of immobilized H4K5,12diac peptides were incubated with increasing amount of ATAD2^342-1390^ for 2 hours prior to the addition of 0.1 μM of recombinant HDAC1 for 2 hours. After stringent washing bound proteins were analyzed in Western blotting. **C.** WT ATAD2 but not ATPase or BD mutant ATAD2 competes with HDAC1 for its interaction with H4K5,12diac. 1 μM of H4K5,12diac peptides were pre-incubated with WT or mutant ATAD2 at given concentration for 2 hours and then incubated with 0.1 μM HDAC1 for additional 2 hours. Bound HDAC1 on the peptides were analyzed in Western blotting.

It has been reported that the new histone marks, H4K5,12diac, are removed by histone deacetylase (HDAC)1, HDAC2 and HDAC3 to allow chromatin compaction during nucleosome assembly [6]. Therefore, we next asked whether ATAD2 may compete with HDAC1 for its interaction with new histone marks. To test this idea, the binding efficiency of HDAC1 and the H4K5,12diac peptides was analyzed by peptide pull down experiment using MCF7 overexpressing either WT or ATPase mutant ATAD2. Indeed, overexpression of WT ATAD2 resulted in less HDAC1 bound on the H4K5,12diac peptides in comparison to ATPase mutant ATAD2 which inefficiently binds to the di-acetylation marks (Figure 7A). Interestingly the same observation was also made to Heterochromatin Protein (HP) 1α. In order to confirm that it is the direct competition between ATAD2 and HDAC1 over the new histone marks we performed *in vitro* binding experiments using recombinant ATAD2 and HDAC1. Clearly increasing amount of recombinant WT ATAD2^342-1390^ outcompeted the interaction of HDAC1 with H4K5,12diac peptides (Figure 7B). Furthermore at similar molar concentration, 0.22 μM, full length WT ATAD2, but neither ATPase mutant nor BD mutant ATAD2 inhibited the association of HDAC1 with the H4K5,12diac peptides (Figure 7C). Hence our results suggest that ATAD2 prevents new histone marks from interacting with HDAC1.

### ATAD2 is associated with heterochromatin replication foci

**Figure 8:**
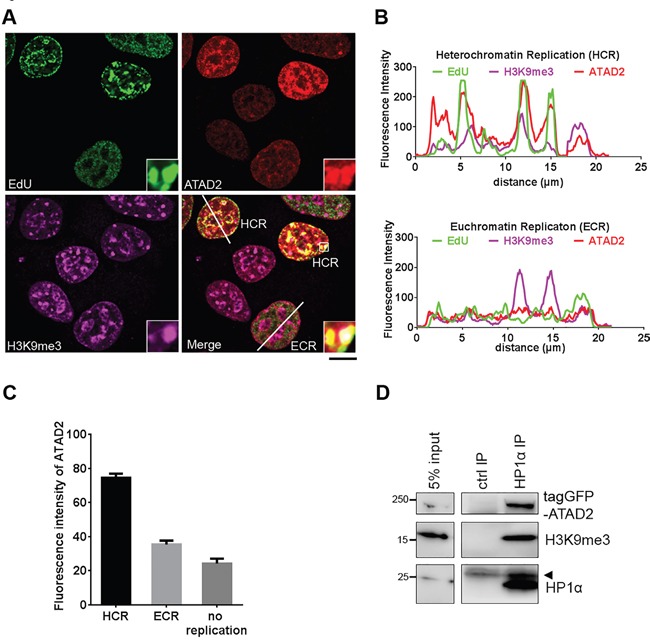
ATAD2 is associated with heterochromatin foci during S phase **A.** and **B.** Localization of ATAD2 on heterochromatin replication foci during S phase. **A.** MCF7 cells were incubated with 10 μM EdU at 37 °C and fixed in 4 % FA for 10 min at RT, followed by direct labeling of EdU using Alexa 488 (green) and indirect staining of ATAD2 (red) and H3K9me3 (purple). Insets show an enlargement of the boxed area by 6 times. (Scale bar: 10 μm) B. Line scan of nucleus of cells during heterochromatin replication (HCR; upper) and euchromatin replication (ECR; lower). Fluorescence intensity along the white line on the merged image (A) are plotted here. **C.** Quantification of fluorescence intensity of ATAD2 in cells undergoing HCR or ECR, or non-replicating cells. HCR was determined by co-localization of replication foci (EdU) and heterochromatin marks (H3K9me3) and ECR was set by no overlapping staining of EdU and H3K9me3 as exemplified in (B). HCR = 74.464 ± 2.447, ECR = 35.374 ± 2.304, no replication = 24.250 ± 2.859. (mean ± SEM., 52 cells for HCR, 42 cells for ECR and 52 cells for ‘no replication’ were used for analysis) **D.** Interaction of ATAD2 and heterochromatin. HP1α associated protein complex was isolated from nuclear extract of MCF7 cells expressing tagGFP-ATAD2 WT by using 2 μg of immobilized HP1α antibodies on Protein G beads and analyzed in Western blotting. Arrow head indicates antibody light chain.

Intrigued by the observation that HP1α was found less on H4K5,12diac peptides in presence of WT ATAD2 in comparison to ATPase mutant (Figure 7A), we hypothesized that ATAD2 might be more relevant for replication of heterochromatin than euchromatin. Supporting this observation we noticed that endogenous ATAD2 (Figure 2A) as well as ectopically expressed ATAD2 (Figure 4A) form speckles in nucleus, similar to that known as pericentric heterochromatin [23]. This observation was more directly assessed by immunofluorescence experiments using antibodies raised against heterochromatin mark, tri-methylation at K9 on histone H3 (H3K9me3). Clearly ATAD2 was found at heterochromatin replication (HCR) foci marked by EdU and H3K9me3 but not at euchromatin replication (ECR) foci (Figure 8A), also confirmed by line scan analysis (Figure 8B). In line with this observation we also found that ATAD2 expression levels were higher in cells undergoing HCR than ECR or non-replicating cells (Figure 8C). In order to further support our findings we employed immunoprecipitation experiments which allow us to directly address whether ATAD2 forms a physical complex with heterochromatin. ATAD2 was co-purified with heterochromatin (Figure 8D) containing HP1α and H3K9me3. Taken together, our data revealed that ATAD2 is associated with heterochromatin replication foci by a physical interaction, perhaps to assist heterochromatin reassembly during replication.

## DISCUSSION

In this study we have discovered a novel function of ATAD2 in DNA replication beyond its previously described role as a transcriptional co-regulator for oncogenes [10, 12–14]. We observed that ATAD2 is predominantly expressed in S phase of the cell cycle in primary tumors and cancer cell lines. While this observation was also reported by Ciro *et al* and Mjelle *et al* previously [14, 24], mechanistic details were not explored. Here we show that ATAD2 is recruited to replication sites by interacting with newly synthesized histones. This mechanism involves direct association of the bromodomain of ATAD2 with di-acetylation mark at K5 and K12 on newly synthesized histone H4 that appears transiently during replication-coupled nucleosome reassembly [25, 26]. It was further supported by cell biological approaches showing that interference of the interaction by mutagenizing the key residues involved in the interaction or depleting newly synthesized histones inhibits recruitment of ATAD2 to replication sites. Through this interaction ATAD2 prevents the new histone marks from interacting with HDAC1. ATAD2 appears more relevant for heterochromatin replication. ATAD2 expression level is increased during heterochromatin replication and it localized to heterochromatin replication foci by a physical interaction with heterochromatin component. This mechanism is important for DNA replication because depletion of ATAD2 or perturbation of ATAD2 BD function by overexpression of dominant negative mutant ATAD2 proteins leads to impaired DNA replication. Thus, our study provides compelling evidence for a general role of ATAD2 in cancer cell proliferation.

Notably, this is the first study that identifies a ‘reader’ of newly synthesized histone H4 di-acetylation marks at K5 and K12. Although this histone modifications are evolutionally conserved from yeast to mammals and known for past decades [5, 26], their function during DNA replication remained largely enigmatic. Our results lead to a new hypothesis how ATAD2 and new histone marks could work in DNA replication. They might regulate the kinetics of nascent chromatin maturation. Higher order chromatin structure formation requires tight regulation of acetylation and deacetylation [27]. Our results suggest that ATAD2 may regulate deacetylation of new histone marks by competing with HDAC1, perhaps to assist proper heterochromatin compaction. ATAD2 was indeed found enriched at heterochromatin replication foci and associated with the heterochromatin component (Figure 8). Previous studies support this possibility. New histone marks have been shown to be more strongly associated with heterochromatin duplication [28] and manipulation of ATAD2 expression levels resulted in altered chromatin compaction in *S. cerevisiae* as well as in embryonic stem cells [29, 30]. Hence, it is tempting to speculate that ATAD2 is initially recruited to nascent chromatin through its interaction with new histone marks and then assists heterochromatin compaction by regulating the acetylation status of new histones.

Whether ATAD2 would be directly involved in structural changes of chromatin by utilizing its ATPase domain as in other chromatin remodelers such as SWI/SNF, ISWI, CHD or INO80 family [31] remains to be explored. However, in our hands purified full length ATAD2 displayed no nucleosome assembly activity in a given *in vitro* condition and the ATPase activity was unaffected in presence of either nucleosomes or DNA (data not shown). It is conceivable that ATAD2 hexamers function as a scaffolding protein to guide building of the higher order chromatin structure by brining nucleosomes into close proximity. This idea is supported by the observation that the monomeric ATAD2 ATPase mutant, is no longer associated with chromatin in spite of the intact bromodomain.

We show the first direct evidence for the importance of the interaction of acetylated histones with a bromodomain protein during DNA replication. Specifically, ATAD2 is the first reader for di-acetylated histone H4 whose role in replication remained unknown for a long time. Such a role for a bromodomain protein outside of transcription regulation is in line with an increasing number of recent studies on roles of histone acetylation in other biological processes beyond transcriptional activation [3, 32, 33]. For instance, recruitment of ZMYND8 to DNA damage site through its association with acetylation on histone H4 is important for DNA damage response [33] and interaction of bromodomain of BRG1 and acetylated histone H3 is essential for DNA double-strand break repair [32].

The general function of ATAD2 in DNA replication proposed here may explain why ATAD2 is highly expressed in many unrelated tumor types [11, 15–19]. Similarly, it predicts that more aggressive tumors such as castration resistant prostate cancer express higher level of ATAD2 compared to primary adenocarcinoma due to higher mitotic index as seen in Supplementary Figure S1A. Hence our work provides an explanation why high levels of ATAD2 are associated with poor prognosis of patients in various cancers.

In conclusion, we describe here a novel function of ATAD2 in DNA replication beyond its transcriptional co-regulator role for oncogenes. Our study provides a plausible reason for the high expression of ATAD2 in higher grade tumors of diverse origin and thereby suggests a potential new therapeutic target to treat aggressive cancers.

## MATERIALS AND METHODS

Cell culture, Primary antibodies, siRNAs, plasmids and transfection, Immunoprecipitation, Immunohistochemistry, Immunofluorescence, Flow cytometry, Fractionation, Fluorescence Recovery After Photobleaching (FRAP), Proteomics, Cloning, Expression and purification of the ATAD2, Isothermal titration calorimetry (ITC), Supplementary Table S1 List of peptides used for TR-FRET experiment, Bioinformatics, Fluorescence polarization (FP), Surface plasmon resonance (SPR), Microscale thermophoresis (MST) are described in Supplemental Experimental Procedures.

### Isolation of proteins on nascent DNA (iPOND)

iPOND was performed as previously described [20]. Briefly, exponentially growing 1 x 10^8^ of HEK 293 cells were incubated with 10 μM EdU (5-ethynyl-2'-deoxyuridine) and fixed in 1 % formaldehyde for 20 min at RT after 20 min (nascent chromatin) or chased for 2 h in fresh medium before fixation (mature chromatin). Crosslinking was stopped by addition of 0.125 M glycine and cells were permeabilized in 0.25 % Triton-X-100 for 30 min at RT followed by Click reaction to couple biotin to EdU. Cells were incubated in Click reaction buffer (0.01 mM biotin azide, 10 mM sodium ascorbate, 2 mM CuSO_4_ in PBS) for 1-2 h at RT with constant mixing. Cells were lysed in lysis buffer (1 % SDS in 50 mM Tris pH 8.0) and chromatin was solubilized by sonication in BIORUPTOR (Diagenode) for 4 min with medium frequency and 0.5 intervals at 4 °C. Biotinylated chromatin fragments were purified on streptavidine-dynabeads (ThermoFisher) by overnight end-over-end rotation at 4 °C. After stringent washing including once with 1 M NaCl the bound chromatin was released and crosslinking was reversed by boiling the beads in 2X LDS buffer for 40 min at 100 °C. Samples were dissolved in NuPAGE Bis-Tris gel and Western blot was performed.

### Time-resolved fluorescence energy transfer (TR-FRET)

TR-FRET measurements of GST-ATAD2 BD^981-1108^ binding to the biotinylated synthetic peptides (Anaspec and Biosyntan) described in Supplementary Table S1 (SI Materials and Methods) were basically carried out as described recently for BRD4. For binding and competition experiments the concentration of GST-ATAD2 was 10 nM. Biotinylated peptides were tested in binding experiments at 100 nM and 1 μM, whereas the biotinylated H4K12ac tracer (HSGRGKGGKGLG-K(Ac)-GGAKRHRK-Biotin) was used at 50 nM in competition assays. In these experiments the unlabeled H4K12ac peptide was titrated at the concentrations indicated in Supplementary Figure S5C. All assays were performed in 50 mM HEPES pH 7.5, 100 mM NaCl, 50 mM KF, 0.25 mM CHAPS, 0.05 % BSA and 1 mM DTT and protein-peptide equilibrium complexes were detected with 10 nM of Anti-GST-XL665 MAb conjugate (Cisbio) and 2.5 nM of SA-Eu3+ Chelate (Perkin Elmer) as TR-FRET acceptor and donor molecules respectively. Raw fluorescence signals were acquired with a Pherastar FS microtiter plate reader (BMG) with 337 nm (excitation), 622 nm (donor emission) and 665 nm (acceptor emission). HTRF® ratio values were calculated as defined in the instrument software and normalized with low and high controls whenever appropriate. Competition experiments were analyzed with a four parameter logistic equation as defined in the GraphPad analysis software (http://www.graphpad.com/guides/prism/6/curve-fitting).

### Peptide pull down

H4 peptides (1-25) conjugated with Biotin was immobilized on Streptavidin-coupled Dynabeads for 2 hours at 4 °C and incubated with nuclear extract from MCF7 cells. Nuclear extract was prepared by first isolating nucleus in hypotonic buffer (10 mM Tris-HCl pH 8.5, 10 mM NaCl, 0.5 mM TCEP and 0.1 % NP-40) and disrupting nuclear envelop in high salt buffer (20 mM Tris-HCl pH 8.5, 420 mM NaCl, 0.5 mM TCEP, 0.1 % Triton X-100, 25 % Glycerol) for 2 hours at 4 °C. Soluble nuclear extract was harvested by centrifugation at 20000 g for 20 min and diluted to 1:3 in buffer without NaCl in order to achieve physiological salt concentration, 140 mM. The nuclear extract was incubated with the immobilized peptides on the Streptavidin-Dynabeads overnight at 4 °C. Next day, the beads were washed three times with washing buffer (20 mM Tris-HCl pH 8.5, 140 mM NaCl, 0.5 mM TCEP, 0.1 % Triton X-100, 25 % Glycerol) and twice with high salt buffer. Bound proteins were recovered by boiling the beads in 1X sample buffer for 5 min. at 95 °C and analyzed by SDS-PAGE and Western Blotting. For direct binding experiments 1 μM of immobilized histone peptides on Streptavidin-Dynabeads were incubated with indicated amounts of ligands for 2 hours at 4 °C. After stringent washing as described above, bound proteins were analyzed in Western Blotting.

## SUPPLEMENTAL EXPERIMENTAL PROCEDURES




