# Supplementary material for: ATAD2 is an epigenetic reader of newly synthesized histone marks during DNA replication
Source: Oncotarget. 2016 Sep 6;7(43):70323–35. doi: 10.18632/oncotarget.11855 (PMC5342555; doi:10.18632/oncotarget.11855)
Supplement: Supplementary file 1 [file oncotarget-07-70323-s001.pdf]

ATAD2 is an epigenetic reader of newly synthesized histone marks during DNA replication

SUPPLEMENTAL EXPERIMENTAL PROCEDURES

Cell culture

MCF7 cells from ATCC were grown in RPMI 1640 medium supplemented with 1E-10M ß-estradiol, 10% FBS Superior (S0615, Biochrom) 2 mU/ml Insulin and 20 mM Glutamine at 37 °C. For SILAC experiment the medium was replaced by SILAC medium (SILAC-K8-R10-Kit, #282986444, Silantes) with supplements mentioned above. To ensure the sufficient labeling, cells were cultured for 3 passages for 2 weeks and labeling efficiency was checked by mass spectrometry. HEK293 cells were grown in medium following suggestions from ATCC.

Primary antibodies, recombinant protein, siRNAs, plasmids and transfection

ATAD2 (ab154139, Abcam; 61369, Active Motif; HPA029424, Sigma), Ki67 (HPA000451, Sigma), TOP2A (HPA026773, Sigma), PCNA (sc-56, Santa Cruz Biotechnology), H4K12ac (07-595, Upstate Millipore), H3 (ab1791, Abcam), EZH2 (5246S, Cell Signalling Technology), GFP (ab290, Abcam), FLAG (F7425, Sigma) and cyclin D1 (sc-718, Santa Cruz Biotechnology), H3K9me3 (ab8898, Abcam), HP1α (ab109028 , Abcam), HDAC1 (ab7028, Abcam) were used for Western blotting, immunofluorescence, flow cytometry, immunohistochemistry and immunoprecipitation. Recombinant HDAC1 protein was purchased from Active Motif. siRNAs against ATAD2 were 5'-GCU ACU GUU UAC UAU CAG GCU-3' [1, 2], 5'-GGU UGU AGC UCC UCC AAA U-3' [3] and 5'- GCUAAGGAUUUCGAGGUAG -3' [3] and control siRNAs were 5'- GCU ACU GUA AUC UAU CAG G-3' and 5'-CAU CGA GGC AGA UAC UAU U-3'. siRNA against PLK1 was 5'-AGA UCA CCC UCC UUA AAU AUU-3'. Full length human ATAD2 was synthesized and inserted into pTagGFP2-C (Evrogen) and ATPase mutant (K473T, E532Q) and BD mutant (V1013A, Y1021A, Y1063A, I1074A) were generated by site-directed mutagenesis. siRNAs and plasmids were introduced by jetPRIME (Polyplus-transfection) according to the manufacturer's recommendations and for FRAP experiment Lipofectamine 3000 (ThermoFisher) was used.

Immunoprecipitation

MCF7 cells in 15 cm dishes were washed twice with ice-cold PBS and collected in Eppendorf tubes. After centrifugation at 300 x g for 3 min at 4 °C IP buffer (40 mM Hepes at pH7.4, 2 mM EDTA, 0.3 % CHAPS) freshly supplemented with protease inhibitor cocktail and phosphoSTOP from Roche was directly added into the cells and incubated for 10 min on ice. Chromatin was then solubilized by sonication in a BIORUPTOR (Diagenode) at medium frequency, 0.5 intervals for 3 min and insoluble material was removed by centrifugation at 20000 x g for 15 min at 4 °C. 2.25 mg of the lysate was incubated with 2 µg of ATAD2 antibody from Sigma and FLAG antibody as a negative control for 1 h by end-over-end rotation followed by additional 1 h incubation with 50 µl of Dynabeads Protein G (10003D, Thermo Fischer Scientific) that were equilibrated and blocked by 1% BSA in IP buffer. The beads were washed 5 times, instantly frozen in liquid N_2 and shipped to Evotec for further analysis.

For immunoprecipitation of a heterochromatin using HP1α antibody nucleus of MCF7 cells expressing tagGFP-tagged WT ATAD2 were isolated by incubating the cells in buffer A (10 mM Tris-HCl pH8.0, 10 mM NaCl, 0.5 mM TCEP (Tris(2-carboxyethyl)phosphine hydrochloride), 0.1 % NP-40, protease inhibitor cocktail) for 5 min on ice and harvesting nucleus after spinning down at 5000 g for 4 min at 4 °C. The nucleus were lysed in IP buffer (20 mM Tris-HCl pH 7.5, 150 mM KCl, 0.5 % NP-40, 2 mM EDTA and protease inhibitor cocktail) for 20 min on ice followed by sonication in a BIORUPTOR (Diagenode) at medium frequency, 0.5 intervals for 3 min. Soluble nuclear extract was harvested by centrifugation at 20000 x g for 15 min at 4 °C and incubated with 2 µg of HP1α or rabbit IgG overnight at 4 °C. Next day equilibrated 50 µl of Protein G Dynabeads (10003D, Thermo Fischer Scientific) was added into the lysate and allowed for additional 2 hrs at 4 °C. The beads were washed 5 times in IP buffer and bound proteins were recovered by cooking in 1X sample buffer for 5 min. at 95 °C and analyzed by SDS-PAGE and Western blotting.

Immunohistochemistry

TMAs were constructed in-house and by Provitro (Berlin, Germany) and Asterand (Detroit, USA) using commercially available tumor samples for which written

informed consent was obtained (Indivumed, Hamburg, Germany; Asterand, Detroit USA; Provitro Berlin, Germany) of different indications. Immunohistochemistry was performed using EnVision System-HRP (DAB) from Dako (K4006 and K4010). Prior to immunostaining the slides were deparaffinized and rehydrated in xylene and graded ethanol to distilled water. Antigen was retrieved by incubating the tissue in boiling Target Retrieval Solution pH 6.0 (Dako S2031) for 40 min and letting them cool down to RT. After quenching endogenous peroxidase in Peroxidase-Block for 15 min at RT the slides were washed twice in washing buffer (PBS with 0.05 % Tween 20) and incubated with primary antibodies in Antibody-Diluent (Dako S2022) for 90 min at RT. Unbound antibodies were washed and the samples were incubated with secondary antibodies conjugated with peroxidase labelled polymer for 60 min at RT. Bound antibodies were then visualized by incubating the slides in DAB reagent for 10 min and the slides were counterstained in hematocylin. Following dehydration in graded ethanol the slides were coverslipped, scanned by a Mirax MIDI slide scanner and analyzed using the Pannoramic viewer software.

Immunofluorescence

Cells were grown on Matrigel-coated coverslips and fixed in 4 % formaldehyde in PBS for 15 min at RT or in 100 % methanol for 7 min at -20 °C. After removing the residual FA or methanol by washing in PBS cells were permeabilized in blocking solution (10 % goat serum and 0.3 % Triton X-100 in PBS) for 10 min at RT and incubated with primary antibodies in blocking solution. Following the removal of unbound antibodies by washing in PBS cells were incubated with secondary antibodies conjugated with Alexa Fluor 488, 555 or 647 (Invitrogen), washed and mounted on slides in presence of DAPI for visualization of nucleus. Images were acquired using an inverted Zeiss LSM700 confocal microscope using a 488 nm Diode-Pumped Solid State Laser with a 63X, 1.3 N.A. oil-immersion objective. For EdU labelling cells were incubated with 10 µM EdU (5-ethynyl-2'-deoxyuridine) for desired time prior to fixation and labeling was performed according to manufacturer's recommendation (Click-iT EdU Alexa Fluor 647 or 488, ThermoFisher). Line scan analysis was carried out using ImageJ.

Flow cytometry

MCF7 cells were harvested in stain buffer (4 % FCS and 0.05 % NaN_3 in PBS) at 5 x 10^6 – 10^7 cells / ml and fixed in 2 % FA for 10 min at RT. For each staining conditions 100 µl of cells were first permeabilized in 100 % MeOH for 10 min at – 20 °C and incubated in stain buffer with 5 % normal goat serum for 10 min at RT for blocking. Then indirect staining was performed by incubating cells with primary antibodies for 1 h at RT followed by labelling with secondary antibodies conjugated with Alexa488 or Alexa647 for 30 min at RT in dark. For analysis of TagGFP-ATAD2 expressing cells GFP was stained by GFP antibody to enhance the fluorescence signal. DNA was stained by DAPI at 1 µg/ml for 10 min at RT. For EdU (5-ethynyl-2'-deoxyuridine) labelling cells were incubated in 10 µM EdU for 2h at 37 °C prior to fixation and staining was carried out according to manufacturer's suggestions (Click-iT® Plus EdU Alexa Fluor® 647 Flow Cytometry Assay Kit, ThermoFisher). Cells were analyzed by flow cytometry using a BD LSR II. All imaging and quantifications were performed using BD FACSDiva Software (BD Biosciences)

Fractionation

Isolation of chromatin bound proteins was carried using Subcellular Protein Fractionation Kit for Cultured Cells (ThermoFisher) according to manufacturer's recommendations. Briefly MCF7 cells grown in 6 well plates after 3 days after knockdown or 16 h after plasmid transfection were washed in cold PBS and harvested. 20 % of cells were saved for whole cell extract and the rest was subjected for subsequent fractionation. Whole cell extract was prepared by incubating cells in ice-cold RIPA buffer (25 mM Tris·HCl pH 7.6, 150 mM NaCl, 1 % NP-40, 1 % sodium deoxycholate, 0.1 % SDS) supplemented with protease inhibitor cocktail (Roche) for 10 min on ice and sonicated at medium power, 0.5 intervals in BIORUPTOR for 3 min at 4 °C. Soluble lysate was recovered by removing insoluble fractions after centrifugation at 20000 x g for 10 min 5 to 20 µg of lysates were used for SDS gel electrophoresis and Western blot.

Fluorescence recovery after photobleaching (FRAP)

FRAP experiment was performed as previously described with minor modifications [4]. MCF7 cells were transfected with TagGFP-tagged WT and mutant ATAD2 using Lipofectamine 3000 (ThermoFisher) and imaged 24 h after transfection. Medium was replaced by DMEM without phenol red complemented with 25 mM Hepes 4 hours after transfection to prevent autofluorescence. Bleaching and image acquisition were performed on an inverted Zeiss LSM700 confocal microscope using a 488 nm Diode-Pumped Solid State Laser with a 40X, 1.3 N.A. oil-immersion objective at 30 °C. A defined area of interest (2.8 µm x 2.8 µm) was photobleached at full laser power and time lapses were taken every 486 msec at 1 % laser power. The image series were imported in Fiji and average fluorescence was in the bleached region $(F(t)_{ROI})$, the total cell nucleus $(F(t)_{total})$ and a background $(F(t)_{BG})$ were determined. The relative fluorescence intensity

($F(t)_{norm}$) was then calculated following equation shown below where F(i) is the mean intensity of a region in the five pre-bleach scans.

$$F(t)_{norm} = ((F(t)_{ROI} - F(t)_{BG})/(F(t)_{total} - F(t)_{BG}) \times ((F(i)_{total} - F(i)_{BG})/(F(i)_{ROI} - F(i)_{BG})$$

The normalized curves were fitted to double exponential curve and half recovery time was calculated using GraphPad Prism 6. For each groups 20 images were analyzed and student *t*-test was used to determine significant differences between the groups.

Proteomics

Sample eluates were pooled according to Figure 2 and subjected to an in-gel digest. Briefly, proteins were reduced with 10 mM dithiothreitol for 30 min, followed by alkylation with 55 mM iodoacetamide for 30 min in the dark. Samples were subjected to a short gel electrophoresis (10 % BisTris gel, MES buffer system) and proteins fixed and stained in the gel. Gels were cut to five slices per sample and proteins digested with trypsin (Promega) overnight.

Peptides were extracted from gel slices and subjected to desalting on C18 Sep-Pak columns (100 mg sorbent weight, Waters). Peptides were eluted with 50% ACN, 0.5% acetic acid, snap-frozen in liquid nitrogen and lyophilized. Samples were loaded onto a reverse phase analytical column (packed in-house with C18 beads), resolved by an acetonitrile gradient using a UPLC system (Thermo Fisher Scientific) and directly electrosprayed via a nanoelectrospray ion source into an LTQ-Orbitrap Velos (Thermo Fisher Scientific). The Orbitrap mass spectrometer was operated in data-dependent acquisition mode to automatically switch between full scans in the orbitrap mass analyzer (R=60.000 at m/z 400) and the acquisition of CID fragmentation spectra (MS/MS mode) of the fifteen most abundant peptide ions in the linear ion trap (LTQ).

All raw files acquired were processed with the MaxQuant software suite (version 1.4.3.20) using the Andromeda search engine for peptide and protein identification and quantification [5, 6]. The experiments were collectively searched against a Swissprot human database (version 03/2014). Carbamidomethylation of cysteine was set as a fixed modification and oxidation of methionine and N-terminal acetylation were set as variable modifications. The minimum required peptide length was seven amino acids and up to two missed cleavages and three labeled amino acids were allowed. A false discovery rate (FDR) of 0.01 was selected for both protein and peptide identifications and a posterior error probability (PEP) less or equal to 0.1 for each peptide-tospectral match was required. The match between runs option was enabled for a time window of 0.5 min.

Contaminants and reverse hits were removed from the list of identified proteins and the logarithm (base 10) of the SILAC ratios were calculated for further statistical analysis. For identification of true binders, the log-ratio between bait-specific and control precipitation of prey i in sample j was modelled as a mixture of two normal distributions:

$$P(xij|+)=\pi TN(xij|\mu j,\sigma j)+(1-\pi T)N(xij|\sigma j)$$

The proportion of true interactors π was set to 0.05. The results did not depend on the exact value. The expected log-ratios for true interactors μj were separately estimated for the twobait-specific precipitates by using the mean of the log-ratios for the bait. The corresponding expected log-ratio for false interactors is 0. The standard deviation was robustly estimated across all negative log-ratios using the first and the second quartile (IQR method).

All raw files acquired were processed with the MaxQuant software suite (version 1.4.3.20) using the Andromeda search engine for peptide and protein identification and quantification [5, 6]. The experiments were collectively searched against a Swissprot human database (version 03/2014). Carbamidomethylation of cysteine was set as a fixed modification and oxidation of methionine, mono-, di- tri- methylation on lysine and arginine, N-terminal acetylation and acetylation on lysine were set as variable modifications. The minimum required peptide length was seven amino acids and up to two missed cleavages and three labeled amino acids were allowed. A false discovery rate (FDR) of 0.01 was selected for both protein and peptide identifications and a posterior error probability (PEP) less than or equal to 0.1 for each peptide-to-spectral match was required. The match between runs option was enabled for a time window of 2 min.

The resulting list of acetylation sites exported from the MaxQuant software was filtered for class I sites (sites which could be localized with high confidence, localization probability $\geq$ 0.75) and used for subsequent statistical and bioinformatical analyses as well as the list of proteins resulting from the proteome analysis.

Cloning, expression and purification of the ATAD2

An ATAD2 synthetic cDNA encoding the BD (residues 981 to 1108) with an N-terminal TEV cleavage site was codon-optimized by Eurofins (Ebersberg, Germany) for expression in Escherichia coli, subcloned into pDONR221 and subsequently recombined with a modified pDEST vector containing a 5 prime GST tag sequence by Gateway cloning (Invitrogen, USA). The protein was overexpressed in *E. coli* BL21 (DE3) induced overnight by 0.1 mM IPTG (isopropyl β-D-thiogalactoside) at 17 °C. The cell pellet from a 20 L

fermenter was re-suspended in 400 mL buffer containing 50 mM HEPES (pH 7.5), 500 mM NaCl, 5% glycerol, 0.5 mM TCEP and protease inhibitor cocktail (ETDA-free). Cells were lysed by using a high pressure homogenizer (Microfluidcs, USA). The lysate was centrifuged at 30000g at 4 °C for 30 min. The GST-fusion protein was purified by GST affinity chromatography using GSH-sepharose (GE Healthcare Life Science). For ITC experiments the GST-tag was removed by TEV cleavage overnight at 4 °C. Both tagged and untagged proteins were further purified by size exclusion chromatography (SEC) using a Superdex 200 26/60 high load column (GE Healthcare Life Science) and a buffer containing 20 mM sodium phosphate (pH 7.5), 50 mM NaCl, 5% glycerol, 0.5 mM TCEP.

cDNA of ATAD2 (residues 342 to 1390) and a BD mutated ATAD2 (residues 342 to 1390; V1013A, Y1921A, Y1063A, I1074A) with an N-terminal TEV cleavage site were codon-optimized for expression in insect cells by Eurofins (Ebersberg, Germany). Both constructs were provided with an N-terminal FLAG tag by Gateway cloning and purified from baculovirus-infected Sf9 cells using anti-FLAG M2 beads (Sigma-Aldrich) and a binding buffer containing 50 mM Tris (pH 8.5), 250 mM NaCl 5% glycerol, 0.5 mM TCEP, complete+EDTA, 0.1% NP40. Proteins were eluted with 150 µg/mL FLAG peptide and further purified by size exclusion chromatography using a Superdex 200 26/60 high load column (GE Healthcare Life Science) and a buffer containing 50 mM Tris (pH 8.5), 250 mM NaCl, 5% glycerol, 0.5 mM TCEP.

After 3 days of expression of FLAG-tagged full length ATAD2 in HEK 293 cells nuclear extract was harvested as described in immunoprecipitation and the recombinant proteins were purified by using anti-FLAG M2 beads (Sigma-Aldrich). Proteins were eluted with 150 µg/mL FLAG peptide and the purity was assessed by SDS-PAGE gel and Coomassie staining.

Isothermal titration calorimetry (ITC)

ITC experiments with ATAD2 BD and H4K12Ac peptide (SGRGKGGKGLG-K(Ac)-GGAKRHRKVLRDNGSGSK-amide) synthesized by Biosyntan (Germany) were performed in SEC buffer (20 mM sodium phosphate, pH 7.5, 50 mM NaCl, 5% glycerol, 0.5 mM TCEP) using an ITC200 system (MicroCal). The ATAD2 protein at a concentration of 26.6 µM was titrated with 200µM H4K12Ac peptide at 25 °C. After an initial injection of 0.2 µL which was not used for data fitting, 19 injections of 2 µL each were performed at 120 s intervals. The protein solution in the sample cell was stirred continuously during the entire experiment at 1000 rpm. Blank titrations of peptide into buffer were performed to correct for heats generated by dilution and mixing. The data were fit to a model corresponding to a single set of binding sites using MicroCal Origin 7.0 (OriginLab).

Bioinformatics

Using cBioPortal genes that exhibited Spearman Score higher than 0.5 were extracted from various indications based on their RNA-seq data [7]. In total 419 samples for prostate adenocarcinoma, 150 samples for prostate metastasis, 960 samples for breast invasive carcinoma, 33 samples for stomach adenocarcinoma, 522 samples for lung adenocarcinoma and 212 samples for colorectal adenocarcinoma were analyzed. Identified positively correlated genes were then examined by using KEGG Pathways in DAVID Bioinformatics Resource 6.7 [8, 9] to identify pathways in which these are enriched. $-\log_{10}(p)$ were plotted as a bar graph.

Fluorescence polarization (FP)

FP analysis of the binding of ATAD2 (a.a. 342-1390) to the H4K12ac peptide (HSGRGKGGKGLG-K(Ac)-GGAKRHRK-TAMRA) was performed in 50 mM HEPES pH 7.5, 10 mM NaCl, 0.001 % Tween 30, 0.015% BSA and 1 mM DTT. For saturation binding experiments ATAD2 was titrated at the concentrations indicated in Fig 5D and Supplementary Figure S5D while keeping a constant concentration of H4K12ac-TAMRA between 3 and 8 nM (>10-fold below K_D). In competition experiments the TAMRA peptide and ATAD2 were used at 50 and 500 nM respectively, while the unlabeled H4K12ac peptide was titrated at the concentrations indicated in Supplementary Figure S5C. Fluorescence anisotropy and polarization signals were acquired with a Tecan Infite M1000 reader at 530 nm excitation and emission 570 nm wavelengths. Binding saturation data were analyzed with a one site, specific binding model and competition curves were fitted to a 4-parameter logistic model using the equations of the same names provided by GraphPad. IC_{50}'s were converted to Ki values using the correction described by Nikolovska-Coleska *et al* [10] for FP competitive binding assays performed under ligand depletion conditions.

Surface plasmon resonance (SPR)

SPR interaction analyses were performed at RT in a Biacore T200 instrument equipped with a CM5 sensor chip (both from GE Healthcare). Typically the sensor chip's first flow cell was used as control surface, and GST-ATAD2 $BD^{981-1108}$ (100 µg/mL in10 mM Acetate buffer pH 5.5) was immobilized at two densities (ranging between 2000 and 10000 RU) on the neighboring flow cells using standard EDC/NHS amine coupling protocols (Biacore Handbook, GE Healthcare). Assays were carried

out in 50 mM HEPES pH 7.5, 100 mM NaCl, 0.001 % v/v Surfactant P20 as running buffer at a flow rate of 30 µl/min. For interaction analysis, serial dilutions of the H4K12ac peptide (at the concentrations indicated in Supplementary Figure S4) were injected for 60 seconds on both control and GST-ATAD2 surfaces. After equilibrium was reached, dissociation in running buffer was followed for 5 minutes. To obtain the kinetic and affinity parameters double-referenced kinetic and steady-state binding responses were respectively fitted to a Langmuir 1:1 interaction model (Biacore™ Assay Handbook) and to a one-site specific binding model using the BiaEval™ analysis software (GE Healthcare).

Microscale thermophoresis (MST)

MST experiments were performed in a Monolith NT.115 instrument (Nanotemper) using premium coated glass capillaries (Nanotemper). GST-ATAD2 BD(amino acid 981-1108) was previously labeled with the fluorescent dye NT-647 using the cysteine reactive Nt-647-Mal labeling kit from Nanotemper, as recommended by the manufacturer. Capillaries were filled with a mixture of 10 µL of labeled GST-ATAD2-BD protein (20 nM) and 10 µL of titrating H4K12ac peptide (at the concentrations indicated in supplementary figure S5A), both in 10 mM HEPES pH 7.5, 150 mM NaCl, 0.05% Tween-20 and 0.5 mg/ml BSA. Thermophoresis measurements were preceded by a "Capillary Scan" procedure, which allowed optimum data acquisition and sample quality control. Data acquisition was carried out with LED intensity and MSP power of 50% and 20% respectively. For manual data analysis 15 seconds "MST on" time were used. Normalized fluorescence values and affinities were calculated as described elsewhere [11] using the analysis software provided with the instrument (Nanotemper).

REFERENCES

1. Zou JX, Revenko AS, Li LB, Gemo AT, Chen HW. ANCCA, an estrogen-regulated AAA+ ATPase coactivator for ERalpha, is required for coregulator occupancy and chromatin modification. Proceedings of the National Academy of Sciences of the United States of America. 2007; 104:18067-18072.
2. Revenko AS, Kalashnikova EV, Gemo AT, Zou JX, Chen HW. Chromatin loading of E2F-MLL complex by cancer-associated coregulator ANCCA via reading a specific histone mark. Molecular and cellular biology. 2010; 30:5260-5272.
3. Caron C, Lestrat C, Marsal S, Escoffier E, Curtet S, Virolle V, Barbry P, Debernardi A, Brambilla C, Brambilla E, Rousseaux S, Khochbin S. Functional characterization of ATAD2 as a new cancer/testis factor and a predictor of poor prognosis in breast and lung cancers. Oncogene. 2010; 29:5171-5181.
4. Philpott M, Rogers CM, Yapp C, Wells C, Lambert JP, Strain-Damerell C, Burgess-Brown NA, Gingras AC, Knapp S, Muller S. Assessing cellular efficacy of bromodomain inhibitors using fluorescence recovery after photobleaching. Epigenetics & chromatin. 2014; 7:14.
5. Cox J, Mann M. MaxQuant enables high peptide identification rates, individualized p.p.b.-range mass accuracies and proteome-wide protein quantification. Nature biotechnology. 2008; 26:1367-1372.
6. Cox J, Neuhauser N, Michalski A, Scheltema RA, Olsen JV, Mann M. Andromeda: a peptide search engine integrated into the MaxQuant environment. Journal of proteome research. 2011; 10:1794-1805.
7. Cerami E, Gao J, Dogrusoz U, Gross BE, Sumer SO, Aksoy BA, Jacobsen A, Byrne CJ, Heuer ML, Larsson E, Antipin Y, Reva B, Goldberg AP, Sander C, Schultz N. The cBio cancer genomics portal: an open platform for exploring multidimensional cancer genomics data. Cancer discovery. 2012; 2:401-404.
8. Huang da W, Sherman BT, Lempicki RA. Systematic and integrative analysis of large gene lists using DAVID bioinformatics resources. Nature protocols. 2009; 4:44-57.
9. Huang da W, Sherman BT, Lempicki RA. Bioinformatics enrichment tools: paths toward the comprehensive functional analysis of large gene lists. Nucleic acids research. 2009; 37:1-13.
10. Nikolovska-Coleska Z, Wang R, Fang X, Pan H, Tomita Y, Li P, Roller PP, Krajewski K, Saito NG, Stuckey JA, Wang S. Development and optimization of a binding assay for the XIAP BIR3 domain using fluorescence polarization. Analytical biochemistry. 2004; 332:261-273.
11. Wienken CJ, Baaske P, Rothbauer U, Braun D, Duhr S. Protein-binding assays in biological liquids using microscale thermophoresis. Nature communications. 2010; 1:100.

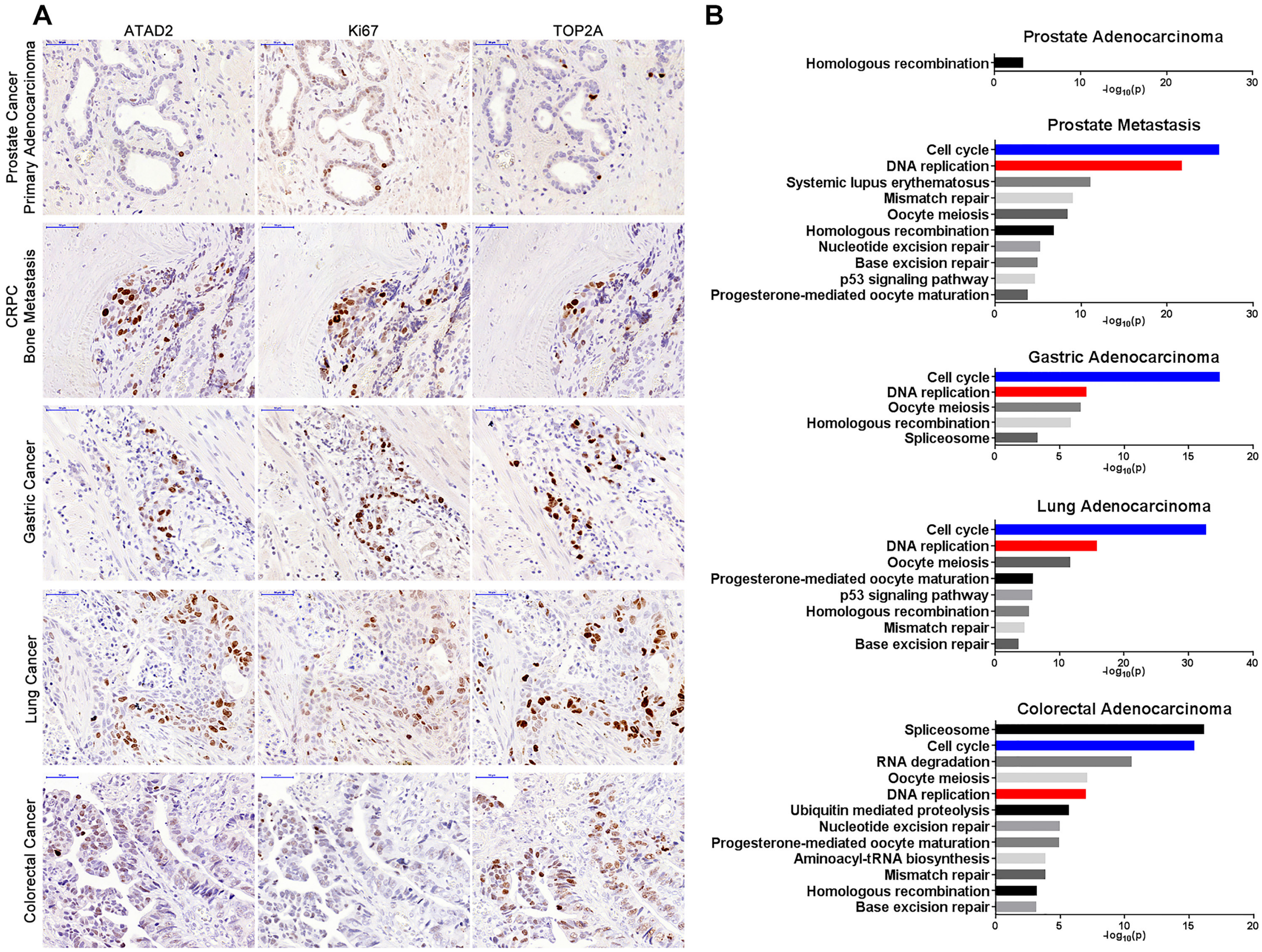

Supplementary Figure S1: Expression of ATAD2 in various tumor types. A. Immunohistochemistry analysis on tissue micro array (TMA) made from prostate (n=35), gastric (n=25), lung (n=45) and colorectal (n=40) cancer samples showed that ATAD2 is expressed in proliferating area of the tumors characterized by positive staining for Ki67 and TOP2A. Shown are representative stainings on actively growing tumor tissue. Stroma and necrotic areas were found negative for ATAD2, Ki67 and TOP2A. **B.** Our oncomining approach found that genes involved in DNA replication (red) and cell cycle (blue) are positively correlated with ATAD2 expression in prostate, gastric, lung and colorectal cancer. $-\log_{10}(p)$ are plotted here. Of note, the reason for oocyte meiosis and mature in tumors from non-reproductive organs is because genes involved in cell cycle and DNA replication have overlapping function in meiosis. 419 samples for prostate adenocarcinoma, 150 samples for prostate metastasis, 33 samples for gastric adenocarcinoma, 522 samples for adenocarcinoma and 212 samples for colorectal adenocarcinoma were taken for analysis.

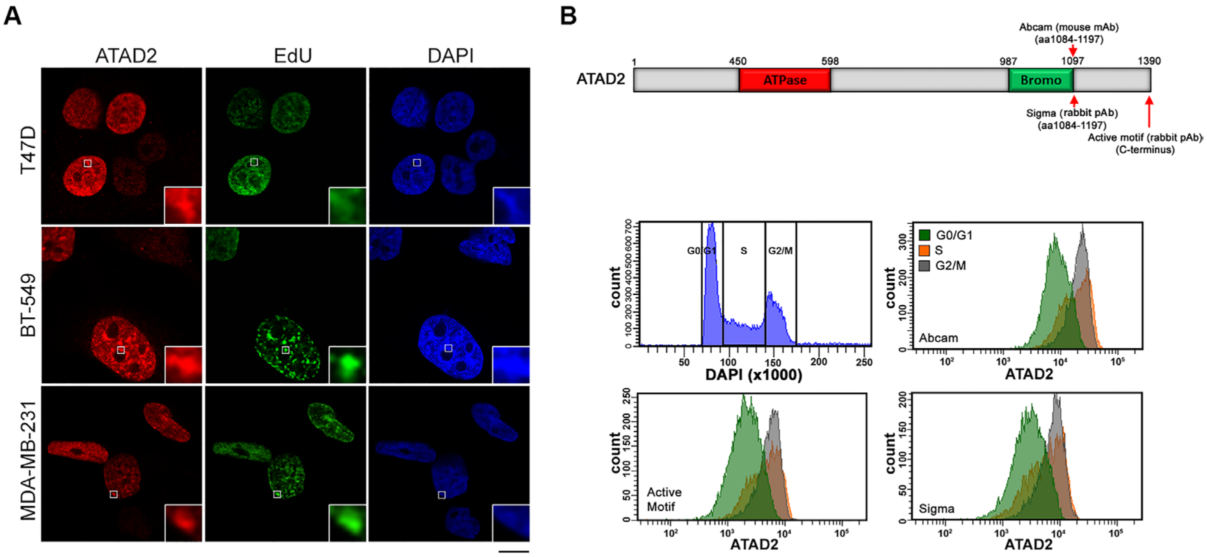

Supplementary Figure S2: ATAD2 is expressed mainly in the S phase of the cell cycle. A. Cells in S phase express ATAD2. T47D, BT-549 and MDA-MB-231 cells were fixed with MeOH and stained for ATAD2 (red). In order to distinguish S phase cells, 10 µM EdU was added into medium for 2 hrs at 37 °C prior to fixation and the EdU was visualized by coupling with Alexa647 (purple). Insets show an enlargement of the boxed area by 6 times. DAPI was used to stain DNA (blue) (scale bar: 10 µm) **B.** Expression analysis by flow cytometry showed ATAD2 expression in S and G2/M phases. Above: a schematic representation of ATAD2 protein shows the position of 3 different ATAD2 antibodies sand their corresponding epitopes. (mAb: monoclonal antibody and pAb: polyclonal antibody) Below: MCF7 cells were fixed in 2 % FA and permeabilized by incubation in 100 % MeOH at -20 °C before staining with ATAD2 antibodies and DAPI. Cells were analyzed in BD LSR II. First, cells were separated by their DNA content (DAPI) into each cell cycle stages and fluorescence intensity of ATAD2 was monitored. Here ATAD2 intensity at different cell cycle stages is plotted.

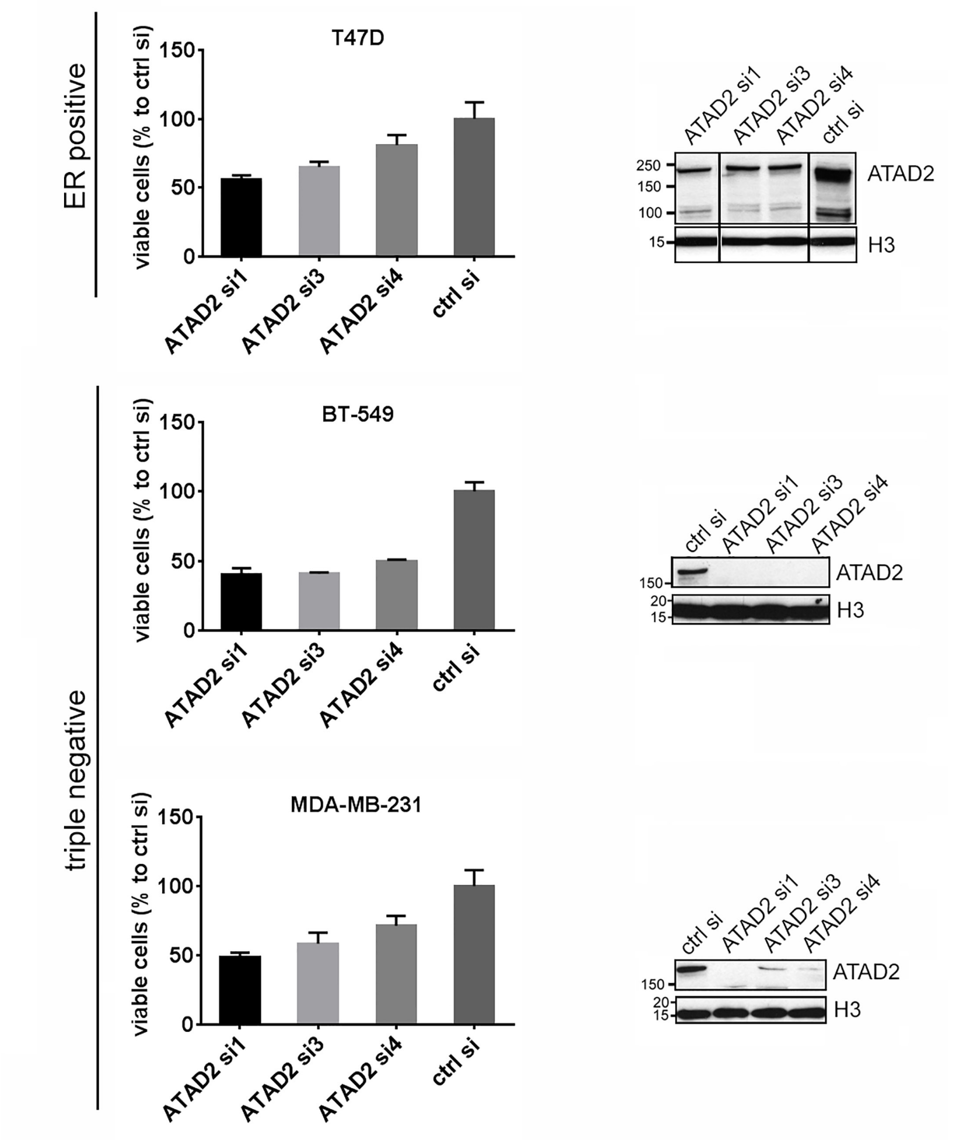

Supplementary Figure S3: Cell viability assay in T47D, BT-549 and MDA-MB-231 upon ATAD2 depletion. Amount of viable cells was measured using AlamarBlue reagent 4 days (BT-549, MDA-MB-231) or 6 days (T47D) after transfection with ATAD2 siRNAs. Fluorescence intensity was normalized to cells transfected with control siRNA (mean ± SD, n = 3). (Right) knockdown efficiency of ATAD2 was monitored by Western blotting. ER: estrogen receptor.

Supplementary Figure S4: list of hits identified in SILAC analysis.

See Supplementary File 1

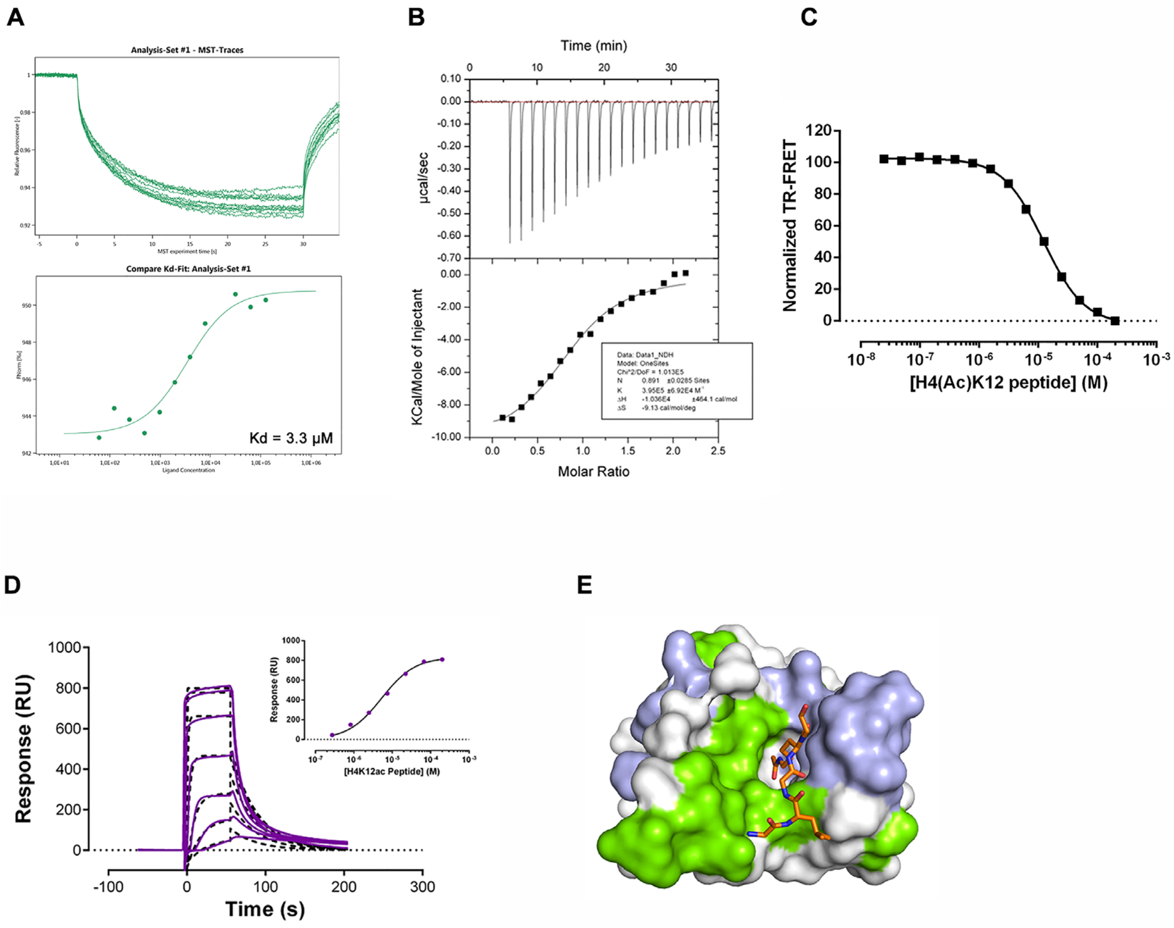

Supplementary Figure S5: Biophysical characterization of H4K12ac recognition by ATAD2 bromodomain (BD). A. MST traces of the interaction of the same peptide and fluorescence labeled GST-ATAD2-BD (upper) and corresponding analysis with a one site specific binding model, which yielded the KD value of 3.3 ± 0.2 µM. **B.** ITC analysis of the interaction of the tag free H4K12ac peptide and ATAD2-BD, from which the following stoichiometry, affinity and thermodynamics parameters of the interaction were derived (N = 0.9, K_D = 2.5 µM, ΔH = -10360 cal/mol, ΔG = -7638 cal/mol -TΔS = 2722 cal/mol. **C.** The indicated concentrations of the unlabeled peptide fully inhibited the interaction of fluorescence labeled H4K12ac peptide and GST-ATAD2-BD (50 and 10 nM respectively) with an IC50 of 12.3 ± 1 µM. **D.** Results obtained with fluorescence based assays were corroborated with "label free" SPR. The sensorgrams (solid lines) resulting from the titration of increasing concentrations of peptide on immobilized GST-ATAD2-BD (2.2 kRU) were fitted to a 1:1 Langmuir model (dotted lines) which delivered a k_{on} = 2.97 ± 0.3 * 10^4 M * s^{-1} and a k_{off} of 3.49 ± 0.3 * 10^{-2} Ms^{-1}, with a K_D = 1.2 ± 0.2 µM. The inset shows a steady state analysis of the data with a one site specific binding model, which yielded an equilibrium dissociation constant (K_D) of 7.9 ± 1.5 µM. **E.** H4K12ac peptide binding site mapping using HSQC NMR data. ^{1}H, ^{15}N labelled ATAD2-BD (50 µM) was titrated with the non-labelled peptide at concentrations between 50 and 250 µM. Concentration-dependent signal perturbations were observed and mapped onto the molecular surface of the ATAD2-BD - Histone H4K12ac peptide structure (PDBcode 4QUT) based on backbone amide resonance assignments. Amino acids displaying resonance perturbations in the presence of the peptide are colored green, residues that were not significantly perturbed are colored white and residues for which backbone assignments are not available are shown in light blue. The H4K12ac peptide is shown in stick representation whereby C, N and O atoms are colored orange, blue and red, respectively.

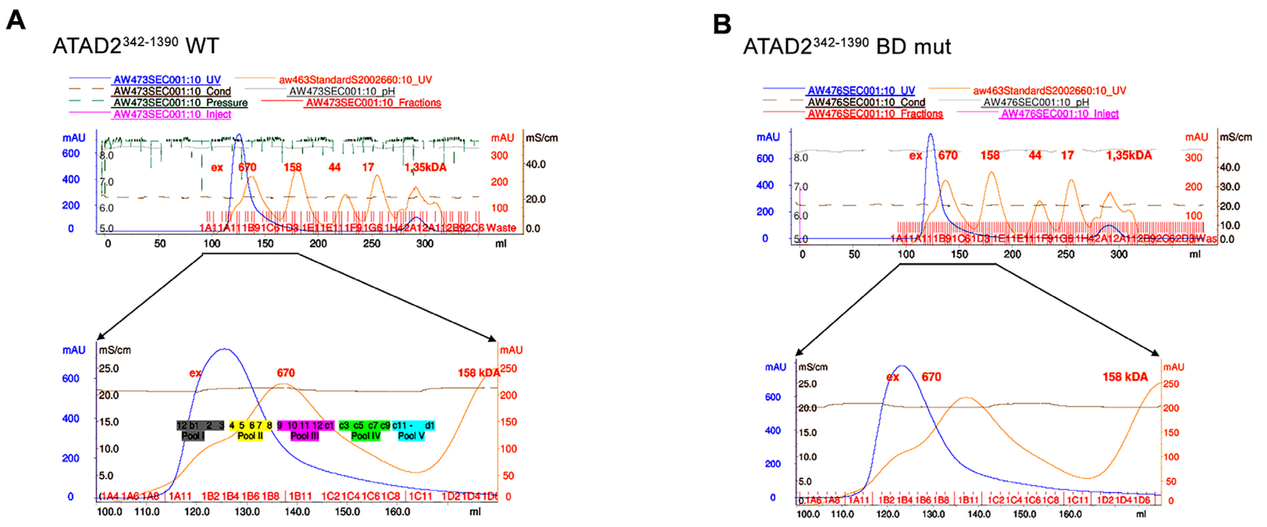

Supplementary Figure S6: Size exclusion chromatography analysis of ATAD$^{2342-1390}$ WT (A) and ATAD$^{2342-1390}$ Bromodomain (BD) mutant (B). The blue traces correspond to the elution pattern of ATAD2$^{342-1390}$ from a gel filtration column and the red traces to the molecular weight standards used under identical chromatographic conditions.

Supplementary Table S1: List of peptides used for TR-FRET experiment

Peptide	Sequence	Producer / cat no.
H1.5 (25-45)	KKAAGAGAAKRKATGPPVSEL-GSGS-K(Biotin)	BIOSYNTAN (Germany)
H1.5 K36ac (25-45)	KKAAGAGAAKR-K(ac)-ATGPPVSEL-GSGS-K(Biotin)	BIOSYNTAN (Germany)
H1.4 (25-45)	GVSLAALKKALAAAGYDVEKN-GSGS-K(Biotin)	BIOSYNTAN (Germany)
H1.4 K33ac (25-45)	GVSLAALK-K(ac)-ALAAAGYDVEKN-GSGS-K(Biotin)	BIOSYNTAN (Germany)
H1.4 (55-75)	KKAGGTPRKASGPPVSELITK-GSGS-K(Biotin)	BIOSYNTAN (Germany)
H1.4 (55-75) K63ac	KKAGGTPR-K(ac)-ASGPPVSELITK-GSGS-K(Biotin)	BIOSYNTAN (Germany)
H2BB (25-45)	DGKKRKRSRKESYSIYVYKVL-GSGS-K(Biotin)	BIOSYNTAN (Germany)
H2BB K34ac (25-45)	DGKKRKRSR-K(ac)-ESYSIYVYKVL-GSGS-K(Biotin)	BIOSYNTAN (Germany)
H2BL (25-45)	DGKKRKRSRKESYSVYVYKVL-GSGS-K(Biotin)	BIOSYNTAN (Germany)
H2BL K34ac (25-45)	DGKKRKRSR-K(ac)-ESYSVYVYKVL-GSGS-K(Biotin)	BIOSYNTAN (Germany)
prelamin A/C (20-40)	PLSPTRITRLQEKEDLQELND-GSGS-K(Biotin)	BIOSYNTAN (Germany)
prelamin A/C K32ac (20-40)	PLSPTRITRLQE-K(ac)-EDLQELND-GSGS-K(Biotin)	BIOSYNTAN (Germany)
lamin-b1 (157-177)	KSLEGDLEDLKDQIAQLEASL-GSGS-K(Biotin)	BIOSYNTAN (Germany)
lamin-b1 K167ac (157-177)	KSLEGDLEDL-K(ac)-DQIAQLEASL-GSGS-K(Biotin)	BIOSYNTAN (Germany)
H4 (70-90)	VTYTEHAKRKTVTAMDVVYAL-GSGS-K(Biotin)	BIOSYNTAN (Germany)
H4K79ac (70-90)	VTYTEHAKR-K(ac)-TVTAMDVVYAL-GSGS-K(Biotin)	BIOSYNTAN (Germany)
H3 (10-30)	STGGKAPRKQLATKAARKSAP-GGK(Biotin)	BIOSYNTAN (Germany)
H3 K18ac (10-30)	STGGKAPR-K(Ac)-QLATKAARKSAP-GGK(Biotin)	BIOSYNTAN (Germany)
H3 K23ac (10-30)	STGGKAPRKQLAT-K(Ac)-AARKSAP-GGK(Biotin)	BIOSYNTAN (Germany)
H3 K18/23ac (10-30)	STGGKAPR-K(Ac)-QLAT-K(Ac)-AARKSAP-GGK(Biotin)	BIOSYNTAN (Germany)
H4 (76-93)	AKRKTVTAMDVVYALKRQ-GSGS-K (Biotin)	BIOSYNTAN (Germany)
H4K79ac (76-93)	AKR-K(ac)-TVTAMDVVYALKRQ-GSGS-K (Biotin)	BIOSYNTAN (Germany)
H4 (76-83)	AKRKTVTA-GSGS-K (Biotin)	BIOSYNTAN (Germany)
H4K79ac (76-83)	AKR-K(ac)-TVTA-GSGS-K (Biotin)	BIOSYNTAN (Germany)
H4 (1-25)	SGRGKGGKGLGKGGAKRHRKVLRDNGSGS - K(Biotin)	AnaSpec Cat. No. 65242-1
H4K5/12ac (1-25)	SGRG - K(Ac) - GGKGLGK(Ac)-GGAKRHRKVLRDNGSGS - K(Biotin)	BIOSYNTAN (Germany)
H4K5/8/12/16ac (1-25)	SGRG - K(Ac) - GGK(Ac)-GLGK(Ac)-GGAK(Ac)-RHRKVLRDNGSGS - K(Biotin)	AnaSpec Cat. No. 65248-1
H4K5ac (1-25)	SGRGKGG - K(Ac) - GLGKGGAKRHRKVLRDNGSGS-K(Biotin)	AnaSpec Cat. No. 65229-1
H4K8ac (1-25)	SGRGKGG - K(Ac) - GLGKGGAKRHRKVLRDNGSGS-K(Biotin)	AnaSpec Cat. No. 65230-1
H4K12ac (1-25)	SGRGKGGKGLG - K(Ac) - GGAKRHRKVLRDNGSGS-K(Biotin)	AnaSpec Cat. No. 65208-1
H4K16ac (1-25)	SGRGKGGKGLGKGGA - K(Ac) - RHRKVLRDNGSGS-K(Biotin)	AnaSpec Cat. No. 65209-1
H3K9,14ac (1-21)	ARTKQTAR - K(Ac) - STGG - K(Ac) - APRKQLA - GGK(Biotin)	AnaSpec Cat. No. 64363-1
H3K9ac (1-21)	ARTKQTAR - K(Ac) - STGGKAPRKQLA - GGK(Biotin)	AnaSpec Cat. No. 64361-1
H3K14ac (1-21)	ARTKQTARKSTGG - K(Ac) - APRKQLA - GGK(Biotin)	AnaSpec Cat. No. 64362-1
