# Supplementary material for: ATAD2 is an epigenetic reader of newly synthesized histone marks during DNA replication
Source: Oncotarget. 2016 Sep 6;7(43):70323–35. doi: 10.18632/oncotarget.11855 (PMC5342555; doi:10.18632/oncotarget.11855)
Supplement: Supplementary file 2 [file oncotarget-07-70323-s002.docx]

**S4 list of hits identified in SILAC analysis.**

| Protein | **Protein names** | **Gene names** | **Position in protein** | **Number of Acetyl (K)** | **Sequence window** | **Acetyl (K) Probabilities** |
| --- | --- | --- | --- | --- | --- | --- |
| Q9UG63 | ATP-binding cassette sub-family F member 2 | ABCF2 | 234 | 1 | GLNGIGKSMLLSAIGKREVPIPEHIDIYHLT | SMLLSAIGK(1)R |
| P23526 | Adenosylhomocysteinase | AHCY | 856 | 1 | THPDKYPVGVHFLPKKLDEAVAEAHLGKLNV | K(1)LDEAVAEAHLGK |
| P04075 | Fructose-bisphosphate aldolase A | ALDOA | 488 | 1 | __MPYQYPALTPEQKKELSDIAHRIVAPGKG | K(1)ELSDIAHR |
| Q86V81 | THO complex subunit 4 | ALYREF | 117 | 1 | ____________MADKMDMSLDDIIKLNRSQ | ADK(1)MDMSLDDIIK |
| P40616 | ADP-ribosylation factor-like protein 1 | ARL1 | 462 | 1 | SKSELVAMLEEEELRKAILVVFANKQDMEQA | K(1)AILVVFANK |
| P25705 | ATP synthase subunit alpha, mitochondrial | ATP5A1 | 35 | 1 | IRADGKISEQSDAKLKEIVTNFLAGFEA___ | LK(1)EIVTNFLAGFEA |
| P27708 | CAD protein;Glutamine-dependent carbamoyl-phosphate synthase;Aspartate carbamoyltransferase;Dihydroorotase | CAD | 285 | 1 | AYLKAMLSTGFKIPKKNILLTIGSYKNKSEL | K(1)NILLTIGSYK |
| P62158 | Calmodulin | CALM1 | 32 | 1 | TIDFPEFLTMMARKMKDTDSEEEIREAFRVF | MK(1)DTDSEEEIR |
| P62158 | Calmodulin | CALM1 | 14 | 1 | TDSEEEIREAFRVFDKDGNGYISAAELRHVM | VFDK(1)DGNGYISAAELR |
| Q9P219 | Protein Daple | CCDC88C | 141 | 1 | TVTEANGKLSQLEFEKRQLHRDLEQAKEKGE | LSQLEFEK(1)R |
| P21926 | CD9 antigen | CD9 | 81 | 1 | LAGGVEQFISDICPKKDVLETFTVKSCPDAI | K(1)DVLETFTVK |
| P23528 | Cofilin-1 | CFL1 | 35 | 1 | KVRKSSTPEEVKKRKKAVLFCLSEDKKNIIL | K(1)AVLFCLSEDKK |
| P23528 | Cofilin-1;Cofilin-2 | CFL1 | 30 | 1 | RYALYDATYETKESKKEDLVFIFWAPESAPL | K(1)EDLVFIFWAPESAPLK |
| O15519 | CASP8 and FADD-like apoptosis regulator;CASP8 and FADD-like apoptosis regulator subunit p43;CASP8 and FADD-like apoptosis regulator subunit p12 | CFLAR | 77 | 1 | EDLDKSDVSSLIFLMKDYMGRGKISKEKSFL | SDVSSLIFLMK(1)DYMGR |
| Q00610 | Clathrin heavy chain 1 | CLTC | 615 | 1 | AISNELFEEAFAIFRKFDVNTSAVQVLIEHI | K(1)FDVNTSAVQVLIEHIGNLDR |
| Q14204 | Cytoplasmic dynein 1 heavy chain 1 | DYNC1H1 | 42 | 1 | IIQERLRYAPLGWSKKYEFGESDLRSACDTV | K(1)YEFGESDLR |
| P68104 | Elongation factor 1-alpha 1;Putative elongation factor 1-alpha-like 3 | EEF1A1 | 114 | 1 | GIGTVPVGRVETGVLKPGMVVTFAPVNVTTE | VETGVLK(1)PGMVVTFAPVNVTTEVK |
| P26641 | Elongation factor 1-gamma | EEF1G | 64 | 1 | EMDECEQALAAEPKAKDPFAHLPKSTFVLDE | AK(1)DPFAHLPK |
| P26641 | Elongation factor 1-gamma | EEF1G | 34 | 1 | REEKQKPQAERKEEKKAAAPAPEEEMDECEQ | K(1)AAAPAPEEEMDECEQALAAEPK |
| P26641 | Elongation factor 1-gamma | EEF1G | 96 | 1 | GEVKLCEKMAQFDAKKFAETQPKKDTPRKEK | K(1)FAETQPK |
| P26641 | Elongation factor 1-gamma | EEF1G | 353 | 1 | LSPDWQVDYESYTWRKLDPGSEETQTLVREY | K(1)LDPGSEETQTLVR |
| P13639 | Elongation factor 2 | EEF2 | 596 | 1 | GLKVRIMGPNYTPGKKEDLYLKPIQRTILMM | K(1)EDLYLKPIQR |
| P63241 | Eukaryotic translation initiation factor 5A-1;Eukaryotic translation initiation factor 5A-1-like;Eukaryotic translation initiation factor 5A-2 | EIF5A | 439 | 1 | HAKVHLVGIDIFTGKKYEDICPSTHNMDVPN | K(1)YEDICPSTHNMDVPNIK |
| P06733 | Alpha-enolase | ENO1 | 61 | 1 | VEHINKTIAPALVSKKLNVTEQEKIDKLMIE | K(1)LNVTEQEK |
| O43559 | Fibroblast growth factor receptor substrate 3 | FRS3 | 37 | 1 | HPARSSDSYAVIDLKKTVAMSNLQRALPRDD | K(1)TVAMSNLQR |
| P50395 | Rab GDP dissociation inhibitor alpha;Rab GDP dissociation inhibitor beta | GDI1 | 86 | 1 | EGIDPKKTTMRDVYKKFDLGQDVIDFTGHAL | K(1)FDLGQDVIDFTGHALALYR |
| P42357 | Histidine ammonia-lyase | HAL | 348 | 1 | EKRVQKSREVIDSIIKEKTVVYGITTGFGKF | EVIDSIIK(1)EK |
| P51610-4 | Host cell factor 1;HCF N-terminal chain 1;HCF N-terminal chain 2;HCF N-terminal chain 3;HCF N-terminal chain 4;HCF N-terminal chain 5;HCF N-terminal chain 6;HCF C-terminal chain 1;HCF C-terminal chain 2;HCF C-terminal chain 3;HCF C-terminal chain 4;HCF C-terminal chain 5;HCF C-terminal chain 6 | HCFC1 | 167 | 1 | SDDDLGTVPDYNQLKKQELQPGTAYKFRVAG | K(1)QELQPGTAYK |
| P16401 | Histone H1.5 | HIST1H1B | 170 | 1 | KKATKKAAGAGAAKRKATGPPVSELITKAVA | K(1)ATGPPVSELITK |
| P10412 | Histone H1.4;Histone H1.3;Histone H1.1;Histone H1t;Histone H1.2 | HIST1H1E | 10 | 1 | AASKERSGVSLAALKKALAAAGYDVEKNNSR | K(1)ALAAAGYDVEK |
| P10412 | Histone H1.4;Histone H1.3;Histone H1.2 | HIST1H1E | 389 | 1 | PVKKKAAKKAGGTPRKASGPPVSELITKAVA | K(1)ASGPPVSELITK |
| P33778 | Histone H2B type 1-B;Histone H2B type 2-E;Histone H2B type 1-O;Histone H2B type 1-J;Histone H2B type 3-B;Putative histone H2B type 2-D;Putative histone H2B type 2-C | HIST1H2BB | 34 | 1 | TKAQKKDGKKRKRSRKESYSIYVYKVLKQVH | K(1)ESYSIYVYK |
| Q99880 | Histone H2B type 1-L;Histone H2B type 1-N;Histone H2B type 1-H;Histone H2B type 1-D;Histone H2B type 1-K;Histone H2B type 1-M;Histone H2B type 2-F;Histone H2B type 1-C/E/F/G/I;Histone H2B type F-S | HIST1H2BL | 96 | 1 | TKAQKKDGKKRKRSRKESYSVYVYKVLKQVH | K(1)ESYSVYVYK |
| P68431 | Histone H3.1;Histone H3.3C;Histone H3.3;Histone H3.2 | HIST1H3A | 541 | 2 | TKQTARKSTGGKAPRKQLATKAARKSAPATG | K(1)QLATK(1)AAR |
| P68431 | Histone H3.1;Histone H3.3C;Histone H3.3;Histone H3.2 | HIST1H3A | 30 | 2 | RKSTGGKAPRKQLATKAARKSAPATGGVKKP | K(1)QLATK(1)AAR |
| P62805 | Histone H4 | HIST1H4A | 277 | 2 | ___MSGRGKGGKGLGKGGAKRHRKVLRDNIQ | GLGK(1)GGAK(1)R |
| P62805 | Histone H4 | HIST1H4A | 253 | 2 | SGRGKGGKGLGKGGAKRHRKVLRDNIQGITK | GLGK(1)GGAK(1)R |
| P62805 | Histone H4 | HIST1H4A | 220 | 1 | NVIRDAVTYTEHAKRKTVTAMDVVYALKRQG | K(1)TVTAMDVVYALK |
| P09429 | High mobility group protein B1;Putative high mobility group protein B1-like 1 | HMGB1 | 401 | 1 | PSAFFLFCSEYRPKIKGEHPGLSIGDVAKKL | IK(1)GEHPGLSIGDVAK |
| P26583 | High mobility group protein B2 | HMGB2 | 1313 | 1 | SYAFFVQTCREEHKKKHPDSSVNFAEFSKKC | K(1)HPDSSVNFAEFSK |
| P07910-2 | Heterogeneous nuclear ribonucleoproteins C1/C2;Heterogeneous nuclear ribonucleoprotein C-like 1 | HNRNPC | 174 | 1 | NSRVFIGNLNTLVVKKSDVEAIFSKYGKIVG | K(1)SDVEAIFSK |
| P08107 | Heat shock 70 kDa protein 1A/1B | HSPA1A | 92 | 1 | NPQNTVFDAKRLIGRKFGDPVVQSDMKHWPF | K(1)FGDPVVQSDMK |
| P11021 | 78 kDa glucose-regulated protein | HSPA5 | 1445 | 1 | TMKPVQKVLEDSDLKKSDIDEIVLVGGSTRI | K(1)SDIDEIVLVGGSTR |
| P04792 | Heat shock protein beta-1 | HSPB1 | 43 | 1 | ERQDEHGYISRCFTRKYTLPPGVDPTQVSSS | K(1)YTLPPGVDPTQVSSSLSPEGTLTVEAPMPK |
| Q92598 | Heat shock protein 105 kDa | HSPH1 | 118 | 1 | RFHGRAFNDPFIQKEKENLSYDLVPLKNGGV | EK(1)ENLSYDLVPLK |
| Q92598 | Heat shock protein 105 kDa | HSPH1 | 158 | 1 | EVMEWMNNVMNAQAKKSLDQDPVVRAQEIKT | K(1)SLDQDPVVR |
| P02545 | Prelamin-A/C;Lamin-A/C | LMNA | 161 | 1 | SSTPLSPTRITRLQEKEDLQELNDRLAVYID | LQEK(1)EDLQELNDR |
| P20700 | Lamin-B1 | LMNB1 | 166 | 1 | ALGDKKSLEGDLEDLKDQIAQLEASLAAAKK | SLEGDLEDLK(1)DQIAQLEASLAAAK |
| P35579 | Myosin-9;Myosin-10;Myosin-14 | MYH9 | 47 | 1 | DHQRQSACNLEKKQKKFDQLLAEEKTISAKY | K(1)FDQLLAEEK |
| P19338 | Nucleolin | NCL | 1897 | 1 | KNDLAVVDVRIGMTRKFGYVDFESAEDLEKA | K(1)FGYVDFESAEDLEK |
| P15559 | NAD(P)H dehydrogenase [quinone] 1 | NQO1 | 56 | 1 | NFNPIISRKDITGKLKDPANFQYPAESVLAY | LK(1)DPANFQYPAESVLAYK |
| O15294 | UDP-N-acetylglucosamine--peptide N-acetylglucosaminyltransferase 110 kDa subunit | OGT | 51 | 1 | LQIVCDWTDYDERMKKLVSIVADQLEKNRLP | K(1)LVSIVADQLEK |
| P62937 | Peptidyl-prolyl cis-trans isomerase A;Peptidyl-prolyl cis-trans isomerase A, N-terminally processed | PPIA | 28 | 1 | EAMERFGSRNGKTSKKITIADCGQLE_____ | K(1)ITIADCGQLE |
| Q06830 | Peroxiredoxin-1 | PRDX1 | 78 | 1 | VDSHFCHLAWVNTPKKQGGLGPMNIPLVSDP | K(1)QGGLGPMNIPLVSDPK |
| P32119 | Peroxiredoxin-2 | PRDX2 | 95 | 1 | VDSQFTHLAWINTPRKEGGLGPLNIPLLADV | K(1)EGGLGPLNIPLLADVTR |
| P78527 | DNA-dependent protein kinase catalytic subunit | PRKDC | 108 | 1 | AAEVLGLILRYVMERKNILEESLCELVAKQL | K(1)NILEESLCELVAK |
| Q15185 | Prostaglandin E synthase 3 | PTGES3 | 47 | 1 | SKHKRTDRSILCCLRKGESGQSWPRLTKERA | K(1)GESGQSWPR |
| Q13882 | Protein-tyrosine kinase 6 | PTK6 | 43 | 1 | NLLHQQMLQSEIQAMKKLRHKHILALYAVVS | DNLLHQQMLQSEIQAMK(1)K |
| Q00765 | Receptor expression-enhancing protein 5 | REEP5 | 75 | 1 | DKAKETADAITKEAKKATVNLLGEEKKST__ | K(1)ATVNLLGEEKK |
| P62906 | 60S ribosomal protein L10a | RPL10A | 115 | 1 | KKLNKNKKLVKKLAKKYDAFLASESLIKQIP | K(1)YDAFLASESLIK |
| P18621 | 60S ribosomal protein L17 | RPL17 | 13 | 1 | AQAKQWGWTQGRWPKKSAEFLLHMLKNAESN | K(1)SAEFLLHMLK |
| P62829 | 60S ribosomal protein L23 | RPL23 | 17 | 1 | MVMATVKKGKPELRKKVHPAVVIRQRKSYRR | K(1)VHPAVVIR |
| P62750 | 60S ribosomal protein L23a | RPL23A | 80 | 1 | VDVKANKHQIKQAVKKLYDIDVAKVNTLIRP | K(1)LYDIDVAK |
| Q9UNX3 | 60S ribosomal protein L26;60S ribosomal protein L26-like 1 | RPL26 | 75 | 1 | KELRQKYNVRSMPIRKDDEVQVVRGHYKGQQ | K(1)DDEVQVVR |
| P46779 | 60S ribosomal protein L28 | RPL28 | 57 | 1 | LKARNSFRYNGLIHRKTVGVEPAADGKGVVV | K(1)TVGVEPAADGK |
| P62888 | 60S ribosomal protein L30 | RPL30 | 9 | 1 | KAKLVILANNCPALRKSEIEYYAMLAKTGVH | K(1)SEIEYYAMLAK |
| P62888 | 60S ribosomal protein L30 | RPL30 | 106 | 1 | _______MVAAKKTKKSLESINSRLQLVMKS | K(1)SLESINSR |
| P61513 | 60S ribosomal protein L37a | RPL37A | 155 | 1 | KYGTRYGASLRKMVKKIEISQHAKYTCSFCG | K(1)IEISQHAK |
| P62424 | 60S ribosomal protein L7a | RPL7A | 3 | 1 | YIRLQRQRAILYKRLKVPPAINQFTQALDRQ | LK(1)VPPAINQFTQALDR |
| P62277 | 40S ribosomal protein S13 | RPS13 | 68 | 1 | LTSDDVKEQIYKLAKKGLTPSQIGVILRDSH | K(1)GLTPSQIGVILR |
| P62269 | 40S ribosomal protein S18 | RPS18 | 4 | 1 | AIKGVGRRYAHVVLRKADIDLTKRAGELTED | K(1)ADIDLTK |
| P62266 | 40S ribosomal protein S23 | RPS23 | 273 | 1 | FIEENDEVLVAGFGRKGHAVGDIPGVRFKVV | K(1)GHAVGDIPGVR |
| P23396 | 40S ribosomal protein S3 | RPS3 | 19 | 1 | ______MAVQISKKRKFVADGIFKAELNEFL | K(1)FVADGIFK |
| P08865 | 40S ribosomal protein SA | RPSA | 24 | 1 | GTNLDFQMEQYIYKRKSDGIYIINLKRTWEK | K(1)SDGIYIINLK |
| Q9Y3B9 | RRP15-like protein | RRP15 | 2334 | 1 | VLNKKTPESKPTILVKNKKLEKEKEKLKQER | PTILVK(1)NK |
| Q9Y6B6 | GTP-binding protein SAR1b | SAR1B | 1074 | 1 | VFLVDCADHERLLESKEELDSLMTDETIANV | LLESK(1)EELDSLMTDETIANVPILILGNK |
| O15047 | Histone-lysine N-methyltransferase SETD1A | SETD1A | 176 | 1 | AKQQAKEEDKEKTKLKEPGLLSLVDWAKSGG | LK(1)EPGLLSLVDWAK |
| P08195 | 4F2 cell-surface antigen heavy chain | SLC3A2 | 93 | 1 | QPGREEGSPLELERLKLEPHEGLLLRFPYAA | LK(1)LEPHEGLLLR |
| Q9UPR3 | Protein SMG5 | SMG5 | 240 | 1 | IVIIPRTVIDGLDLLKKEHPGARDGIRYLEA | TVIDGLDLLK(1)K |
| O75643 | U5 small nuclear ribonucleoprotein 200 kDa helicase | SNRNP200 | 4204 | 1 | RQLSRFYDDAIVSQKKADEVLEILKTASDDR | K(1)ADEVLEILK |
| Q8N5J4 | Transcription factor Spi-C | SPIC | 79 | 1 | QFVSKNKEKLAELWGKRKGNRKTMTYQKMAR | LAELWGK(1)R |
| P37108 | Signal recognition particle 14 kDa protein | SRP14 | 1169 | 1 | ITLKKYDGRTKPIPKKGTVEGFEPADNKCLL | K(1)GTVEGFEPADNK |
| P63313 | Thymosin beta-10 | TMSB10 | 4 | 1 | ____________MADKPDMGEIASFDKAKLK | ADK(1)PDMGEIASFDK |
| O14656 | Torsin-1A | TOR1A | 156 | 1 | LDFWRSGKQREDIKLKDIEHALSVSVFNNKN | LK(1)DIEHALSVSVFNNK |
| P60174 | Triosephosphate isomerase | TPI1 | 84 | 1 | SRKFFVGGNWKMNGRKQSLGELIGTLNAAKV | K(1)QSLGELIGTLNAAK |
| Q15643 | Thyroid receptor-interacting protein 11 | TRIP11 | 772 | 1 | MFRETIQNLSRIIREKDIEIDALSQKCQTLL | EK(1)DIEIDALSQK |
| P43897-3 | Elongation factor Ts, mitochondrial | TSFM | 613 | 2 | MMHCQTLKDQPSAYSKENWEKT_________ | DQPSAYSK(1)ENWEK(1)T |
| P43897-3 | Elongation factor Ts, mitochondrial | TSFM | 133 | 2 | TLKDQPSAYSKENWEKT______________ | DQPSAYSK(1)ENWEK(1)T |
| P10599 | Thioredoxin | TXN | 896 | 1 | KKGQKVGEFSGANKEKLEATINELV______ | EK(1)LEATINELV |
| P12956 | X-ray repair cross-complementing protein 6 | XRCC6 | 114 | 1 | LKEACRAYGLKSGLKKQELLEALTKHFQD__ | K(1)QELLEALTK |
| P63104 | 14-3-3 protein zeta/delta | YWHAZ | 112 | 1 | _____________MDKNELVQKAKLAEQAER | MDK(1)NELVQK |
